# Claudin 6 is a suitable target for CAR T-cell therapy in atypical teratoid/rhabdoid brain tumors and other pediatric solid tumors

**DOI:** 10.1136/jitc-2025-011709

**Published:** 2025-10-10

**Authors:** Peter J Madsen, Anna Melissa Schlitter, Carina Flemmig, Conor Dickson, Kyra Harvey, Cullen Wilson, Ezra Beaubien, Luke Patterson, Allison Stern, Crystal Griffin, Nikhil Joshi, Sreehita Hajeebu, Daniel Martinez, Phillip B Storm, Adam C Resnick, Peter Hillemanns, Martin Stanulla, Jörg Faber, Arthur Wingerter, Matthias Martin Gaida, Saskia Holtemeyer, Mark Laible, Anja Feldner, Florian Frohns, João H Duarte, Bruno Valentin Sinn, Stefan Wöll, Ugur Sahin, Özlem Türeci, Jessica B Foster

**Affiliations:** 1 Division of Neurosurgery, Children's Hospital of Philadelphia, Philadelphia, PA, USA; 2 Department of Neurosurgery, Perelman School of Medicine, University of Pennsylvania, Philadelphia, PA, USA; 3 Center for Data Driven Discovery in Biomedicine (D3b), Children’s Hospital of Philadelphia, Philadelphia, PA, USA; 4 BioNTech SE, Mainz, Germany; 5 TUM School of Medicine and Health, Technical University of Munich, Munich, Germany; 6 Division of Oncology and Center for Childhood Cancer Research, Children's Hospital of Philadelphia, Philadelphia, PA, USA; 7 Pathology and Laboratory Medicine, Perelman School of Medicine, University of Pennsylvania, Philadelphia, PA, USA; 8 Departments of Gynecology and Obstetrics, and Comprehensive Cancer Center, Hannover Medical School, Hannover, Germany; 9 Department of Paediatric Haematology and Oncology, Hannover Medical School, Hannover, Germany; 10 Department of Pediatric Hematology/Oncology/Hemostaseology, Center for Pediatric and Adolescent Medicine, University Medical Center, Johannes Gutenberg University Mainz, Mainz, Germany; 11 University Cancer Center Mainz (UCT Mainz), University Medical Center, Johannes Gutenberg University Mainz, Mainz, Germany; 12 Institute of Pathology, University Medical Center, Johannes Gutenberg University Mainz, Mainz, Germany; 13 Translational Oncology (TRON gGmbH) at the University Medical Center, Johannes Gutenberg University Mainz, Mainz, Germany; 14 Research Center for Immunotherapy, University Medical Center, Johannes Gutenberg University Mainz, Mainz, Germany; 15 BioNTech Cell and Gene Therapies GmbH, Mainz, Germany; 16 Helmholtz-Institute for Translational Oncology Mainz (HI-TRON Mainz), German Cancer Research Center (DKFZ), Mainz, Germany; 17 Department of Pediatrics, Perelman School of Medicine, University of Pennsylvania, Philadelphia, PA, USA

**Keywords:** Chimeric antigen receptor - CAR, Solid tumor, Biomarker, Central Nervous System Cancer

## Abstract

**Background:**

Solid tumors comprise approximately 60% of all pediatric cancers. Relapsed or refractory tumors of the central nervous system (CNS), such as atypical teratoid/rhabdoid tumors (AT/RTs), are the leading cause of death in children with cancer. Claudin 6 (CLDN6)-specific chimeric antigen receptor (CAR) T cells have demonstrated activity in preclinical and clinical studies in various solid adult cancers. However, the suitability of CLDN6 as a target in pediatric tumors and their susceptibility to CAR T-cell therapy has yet to be established. This study aimed to evaluate the suitability of CLDN6 as a target for CAR T-cell therapy of pediatric solid tumors.

**Methods:**

Immunohistochemical CLDN6 expression was assessed in fetal normal tissues (n=91), pediatric normal tissues (n=157), and two sets of pediatric tumor tissues (n=527 and n=49) using a combined score that includes the percentage of stained cells with a 4-point intensity scale (0 to 3+). The antitumor activity of CLDN6 RNA-transduced CAR T cells against AT/RT cell lines was assessed with in vitro assays and in immunodeficient NOD-SCID-γc–/– (NSG) mouse models bearing orthotopic xenograft tumors.

**Results:**

Membranous CLDN6 expression, as detected by immunohistochemistry, was widely observed in fetal tissues but was absent in almost all non-malignant pediatric tissues, except for very rare, scattered cells with 1+ to 2+ intensity in kidney, pancreas, pituitary, and salivary gland tissues. Membranous CLDN6 expression was frequently detected in a subset of the pediatric tumor entities, including germ cell tumors (93% of samples with CLDN6-positive cells), nephroblastoma (64%), extracranial malignant rhabdoid tumors (50%), and AT/RTs (39%). In CLDN6-positive samples, CLDN6 was generally expressed with 2+ or 3+ intensity in substantial proportions of the cancer cells. Strong CLDN6 expression was also detected in single samples of hepatoblastoma, Ewing sarcoma/other embryonal tumors, and osteosarcoma.

In experimental models, CLDN6-CAR T cells led to antigen-specific killing of endogenously CLDN6-expressing AT/RT cell lines in vitro and exhibited potent and specific antitumor activity in mice bearing orthotopic CLDN6-expressing AT/RT xenograft tumors.

**Conclusions:**

These results support CLDN6 as an oncofetal cell-surface antigen that may be suitable for CAR T-cell targeting in pediatric solid tumors, including those of the CNS.

**WHAT IS ALREADY KNOWN ON THIS TOPIC**

- Chimeric antigen receptor (CAR) T-cell therapy may be feasible for pediatric solid tumors, including brain tumors, and claudin 6 (CLDN6)-specific CAR T cells have shown promising clinical activity in adult patients with CLDN6-positive solid tumors. However, CLDN6 expression in pediatric tumors and normal tissue has not been comprehensively investigated.

**WHAT THIS STUDY ADDS**

- Through semiquantitative immunohistochemical analysis of large sample sets of pediatric normal and tumor tissues, this study demonstrates that membranous CLDN6 expression is absent in the vast majority of normal organs but frequent in several pediatric solid tumors with high medical need, including germ cell tumors, nephroblastoma, extracranial malignant rhabdoid tumors, and atypical teratoid/rhabdoid tumors. Additionally, in vitro and in vivo experiments demonstrate that CLDN6-expressing atypical teratoid/rhabdoid pediatric tumors are susceptible to a CAR T-cell therapeutic approach.

**HOW THIS STUDY MIGHT AFFECT RESEARCH, PRACTICE OR POLICY**

- These results support further studies of CLDN6-targeted CAR T cells as a potential novel therapy for hard-to-treat pediatric solid tumors, including those of the central nervous system.

## Background

 Over 400,000 children and adolescents are diagnosed with cancer each year,^1^ with solid tumors accounting for approximately 60% of cases.^2^ Relapsed or refractory tumors of the central nervous system (CNS) are the leading cause of death in children with cancer.^3 4^ Pediatric patients with atypical teratoid/rhabdoid tumors (AT/RTs), particularly those with high-risk disease, have a dismal prognosis despite intensive multimodal treatments.^5^,^7^

Recent studies have demonstrated the feasibility of chimeric antigen receptor (CAR) T-cell therapy for pediatric solid tumors, including brain tumors, with encouraging efficacy.^8^,^12^ However, the overall success of CAR T-cell and other potent targeted therapies for solid tumors remains limited, in part due to a lack of antigens selectively expressed on tumor-cell surfaces but not on normal cells.^13 14^

Claudin 6 (CLDN6) is a primitive oncofetal cell-surface antigen that is physiologically expressed during organogenesis, silenced in adult normal tissues, but may escape transcriptional silencing in the course of malignant transformation, leading to its aberrant expression in several adult^15^,^20^ and pediatric^21^ tumor entities.

Due to its exquisite cancer cell selectivity, CLDN6 has been proposed and investigated as a therapeutic target.^18 22^ Several modalities targeting CLDN6 in adult tumors have now entered clinical testing, including CAR T cells and bispecific T-cell engagers (NCT05410717, NCT04503278, NCT05317078, NCT05735366, NCT05394675, NCT05103683).

CLDN6-specific second-generation CAR T cells, in combination with a CAR T cell-amplifying RNA vaccine (CARVac), have been shown to mediate the elimination of CLDN6-expressing tumors in xenograft and syngeneic mouse models.^23^ They are currently being tested in adult patients with heavily pretreated relapsed/refractory CLDN6-positive solid tumors in a phase 1 clinical trial for safety and feasibility (NCT04503278). Results from the ongoing trial show manageable toxicity, robust CAR T-cell engraftment, well-tolerated combination with CARVac, and promising clinical activity.^24^,^26^

Currently, the suitability of CLDN6 CAR T cells for pediatric patients remains uncertain. Research on cell-surface expression of CLDN6 in pediatric normal tissues has been limited,^27 28^ and additional data is needed to confirm the absence of CLDN6 expression in vital organs after birth.

Cell-surface expression of CLDN6 in pediatric tumors has been investigated, but less comprehensively than in adult tumors so that potential patient populations eligible for CLDN6-targeted treatment have not been determined, and conclusions regarding CLDN6 expression in AT/RTs differ.^2127^,^30^

## Objectives

The objectives of this study were twofold. First, we sought to evaluate the suitability of CLDN6 as a therapeutic target for pediatric solid tumors by characterizing CLDN6 expression by immunohistochemical (IHC) analysis in a broad sample set of fetal as well as pediatric normal tissues and organs from different age groups from birth to adolescence, and pediatric tumor tissues with a focus on solid tumors. Second, we evaluated the susceptibility of CLDN6-expressing pediatric AT/RT brain tumors to CLDN6-targeted CAR T-cell killing, using in vitro assays and orthotopic mouse models.

## Results

### CLDN6 is expressed in fetal tissues but is largely absent in pediatric tissues

**Figure 1:**
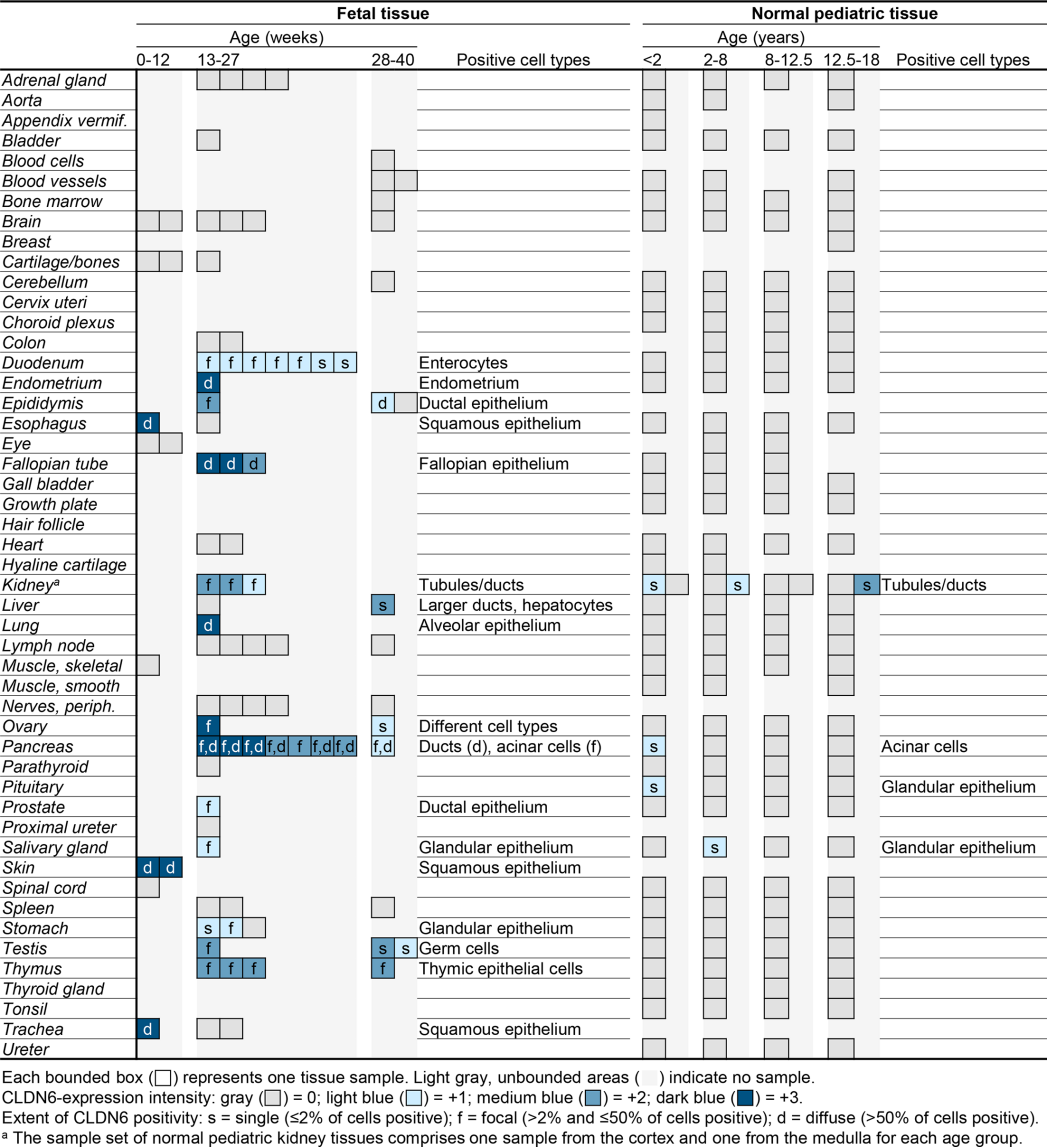
CLDN6 is expressed in a subset of fetal tissues but is absent in healthy pediatric tissues and organs with rare exceptions. The expression of CLDN6 protein in fetal and pediatric normal tissues was determined by semiquantitative immunohistochemistry assay. A board-certified pathologist evaluated samples for both the predominant membranous CLDN6-staining intensity and the percentage of CLDN6-positive cells per tissue sample. Each investigated tissue is represented by a bounded box and is classified according to tissue type and the age of the donor. The staining intensity of each tissue type was scored using a 4-point scale: negative (0, gray), weakly positive (1+, light blue), medium positive (2+, blue), and strongly positive (3+, dark blue). The percentage of CLDN6-positive cells was classified as single (s, ≤2% positive), focal (f, >2% and ≤50% positive), and diffuse (d, >50% positive). CLDN6, claudin 6.

**Figure 2:**
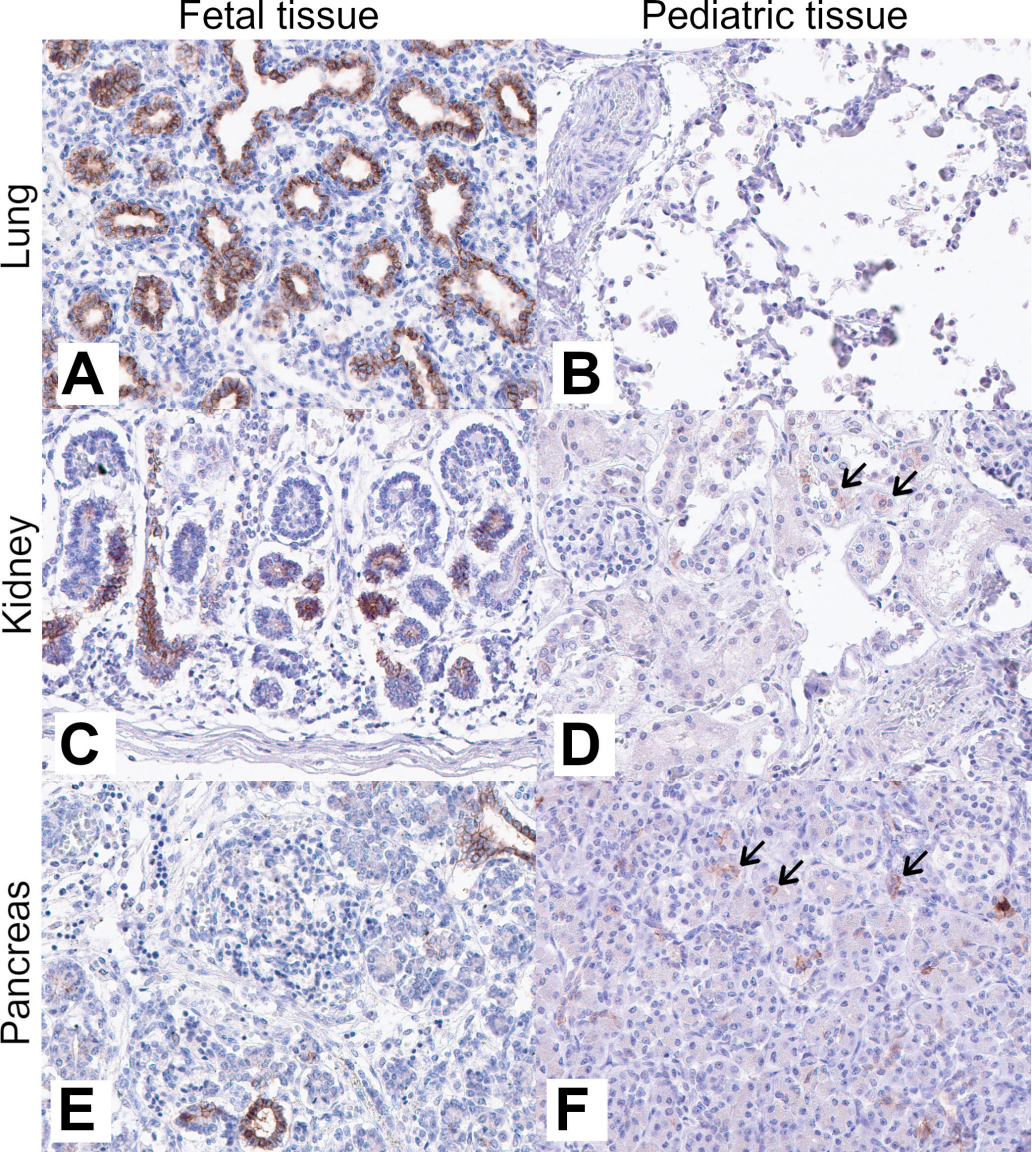
CLDN6 is expressed in a subset of fetal tissues but is largely downregulated or completely absent after birth. Immunohistochemical expression analysis of CLDN6 in fetal and pediatric tissues. (A) Fetal lung (20+4 weeks) and (B) lung tissue on day 3 postnatal. (C) Fetal kidney (20+4 weeks) and (D) kidney tissue at 15 months postnatal. (E) Fetal pancreas (17 weeks) and (F) pancreas tissue on day 12 postnatal. Brown color indicates the presence of CLDN6 antigen; blue color is a hematoxylin counterstain. Arrows indicate single positive cells with membranous CLDN6 expression in pediatric kidney and pancreas tissue. Focal non-specific background positivity can be seen in postnatal pancreatic tissue (F). A 20× objective was used to capture images of digital slides. CLDN6, claudin 6.

Previous studies have observed CLDN6 expression in circulating fetal cells by stem cell microarray analysis,^31^ in fetal stomach, pancreas, lung, and kidney tissues by quantitative real-time PCR,^23^ and membranous expression in fetal liver, lung, kidney, and heart by IHC.^28^ We first set out to more comprehensively characterize the membranous expression of CLDN6 pre-birth by analyzing 91 samples from 35 fetal tissues from all three trimesters of pregnancy using IHC CLDN6 expression analysis (figures1  2). We screened tissue for the presence of CLDN6-positive cells and assessed their membranous staining intensity using a 4-point intensity scale: negative (0), weakly positive (1+), medium positive (2+), and strongly positive (3+). Additionally, we noted the specific cell type and the extent of CLDN6 positivity within that cell type (single cells (≤2%), focal (>2% and ≤50%) or diffuse (>50%)). Membranous CLDN6 expression was detected in 17 of the 35 (49%) tested fetal tissue types, mainly in epithelial cell types, including fetal alveolar epithelium, esophageal epithelium, epidermis, acinar and ductal cells of the pancreas, and renal tubules. Whereas homogenous and strongly positive expression was observed in many epithelial tissues (eg, fetal alveolar epithelium, see figure 2), only focal and weakly to medium positive CLDN6 expression was observed in testicular germ cells. No CLDN6 expression was observed in cartilage, bones, vessels, and skeletal muscle. CLDN6 expression was observed in tissues from all trimesters of pregnancy, but strongly positive CLDN6 expression was only observed in the first and second trimesters (figure 1).

We next sought to assess whether CLDN6 expression is silenced in non-cancerous, normal pediatric tissues, as has been demonstrated in normal adult tissues.^23^ To this end, we analyzed 157 tissue samples from 42 normal pediatric tissue types across four age groups ranging from birth to 18 years of age as described above (figures1  2). In contrast to fetal tissue, the vast majority of normal pediatric tissue types analyzed (38/42) showed no CLDN6 staining in any age group, including tissue types that expressed CLDN6 during fetal development (eg, alveolar epithelium; see figure 2). CLDN6 expression in the remaining samples was limited to scattered single cells in the kidney (3/8 samples), pancreas (1/4 samples), pituitary (1/4 samples), and salivary gland (1/4 samples). Compared with during fetal development, CLDN6 expression was largely downregulated in pediatric kidney and pancreas tissues. Specifically, focal CLDN6 expression was observed in tubules and ducts with low to moderate intensity in all fetal kidney samples, but only three out of eight pediatric samples (ranging from 0 to 18 years) showed single cells with CLDN6 positivity, at weak to medium intensity. Similarly, CLDN6-positive cells were observed in only one out of four pediatric pancreatic tissues, whereas all fetal pancreatic tissues showed focal to diffuse CLDN6 positivity with medium to strong intensity. Likewise, only one out of four pediatric salivary gland samples showed single cells with CLDN6 positivity at weak intensity. Notably, five of these six samples exhibiting CLDN6-positive cells were from children aged 0–8 years, while the sixth positive sample (kidney) was in the 12.5–18 years age group. Taken together, the results show that membranous CLDN6 expression is broadly present in several fetal normal tissue types and is almost completely absent in normal pediatric tissues and organs.

### CLDN6 is highly expressed in a subset of solid pediatric tumor entities

**Figure 3:**
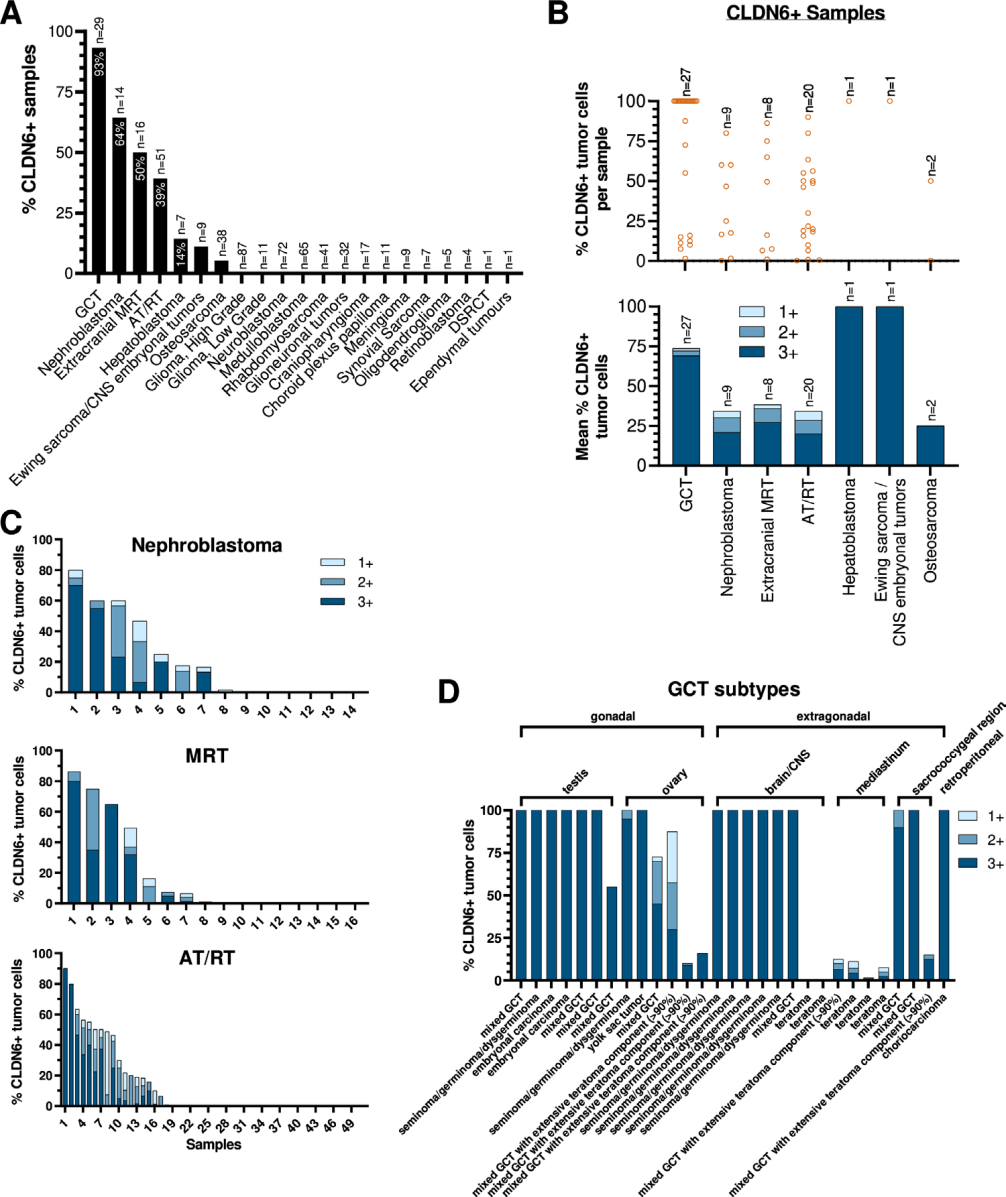
CLDN6 is robustly expressed in a subset of solid pediatric tumor entities. CLDN6 protein expression in pediatric tumor entities, as determined by semiquantitative immunohistochemistry assay and assessed for negative (0), weakly positive (1+), medium positive (2+), and strongly positive (3+) membranous staining intensity. (A) Proportion of samples per tumor entity with any CLDN6-positive (CLDN6+) cells. (B) CLDN6 expression in samples with CLDN6-positive cells. (Top) Proportion of cells per sample that stained positive for CLDN6, with any staining intensity. (Bottom) Mean proportions of CLDN6-positive cells per tumor entity, broken down by staining intensity. (C) Proportions of CLDN6-positive cells per sample in nephroblastoma, AT/RT, and MRT, broken down by staining intensity. (D) Proportions of CLDN6-positive cells per sample in GCT subtypes. n=number of tissue samples; each sample originates from an individual patient. AT/RT, atypical teratoid/rhabdoid tumor; CLDN6, claudin 6; CNS, central nervous system; DSRCT, desmoplastic small round cell tumor, GCT, germ cell tumor; MRT, malignant rhabdoid tumor.

To gain a more complete picture of the aberrant membranous protein expression of CLDN6 in pediatric cancers, 527 tissue samples from a broad set of 21 pediatric tumor entities were analyzed by semiquantitative IHC. Tumor samples were wide-ranging and inclusive of the most common solid tumors encountered in pediatrics, as well as multiple rare tumor types. For the majority of the tumor entities, representative sample sizes were available, whereas other tumor entities were represented by only one sample from a single patient. CLDN6 expression was observed in at least one tissue sample from 7 of the 21 tumor entities (figures3A  4; online supplemental table 1). Expression was most frequent in germ cell tumors (GCTs) from all different locations including intracranial GCTs (27/29, (93%) of samples with any CLDN6 expression), followed by nephroblastoma (9/14, 64%), and extracranial malignant rhabdoid tumors (MRTs; 8/16, 50%). AT/RTs were also frequently CLDN6-positive (20/51, 39%), whereas all other investigated childhood brain tumors did not express CLDN6 (see online supplemental table 1). Further, CLDN6 expression was found in individual cases of hepatoblastoma, Ewing sarcoma/other embryonal tumors, and osteosarcoma.

**Figure 4:**
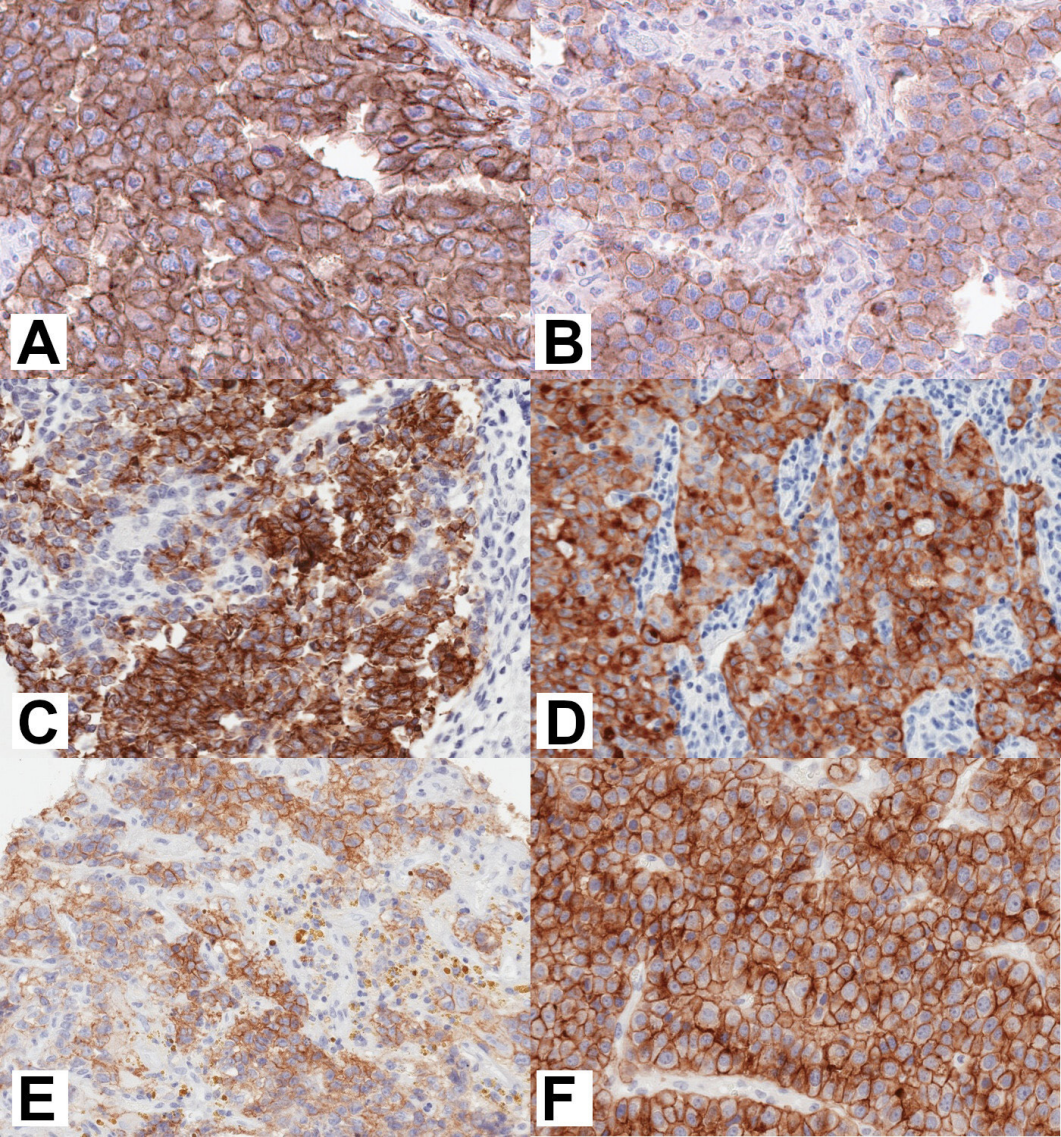
Strong membranous CLDN6 expression is found in several pediatric solid tumor entities. Immunohistochemical expression analysis of CLDN6 in different tumor tissues. (A) Primary GCT of the testis (seminoma), (B) primary GCT of the ovary (germinoma), (C) primary nephroblastoma, (D) lymph node metastasis of extracranial malignant rhabdoid tumor, (E) cerebral AT/RT and (F) primary hepatoblastoma. Brown color indicates the presence of CLDN6 antigen, blue background is a hematoxylin counterstain. All cases show a distinct moderate to strong membranous CLDN6 expression. The 20× objective was used to capture images of digital slides. AT/RT, atypical teratoid/rhabdoid tumor; CLDN6, claudin 6; GCT, germ cell tumor.

Intratumor homogeneity of CLDN6 expression varied between tumor entities. GCTs were highly homogenous with regard to the intensity of CLDN6 expression and the relative proportion of CLDN6-positive tumor cells per sample. For the 27 CLDN6-positive GCT samples, the mean proportion of CLDN6-positive tumor cells was 74%, of which 69% were strongly positive (3+) (figure 3B, online supplemental table 1). CLDN6 expression was more heterogeneous in extracranial MRTs, AT/RTs, and nephroblastomas with regard to the proportion of CLDN6-positive cells per sample, but was overwhelmingly either medium (2+) or strongly positive (3+) (figure 3B and C; figure 4; online supplemental table 1). Of note, 100% of tumor cells in the single positive samples of hepatoblastoma (see figure 4) and Ewing sarcoma/other embryonal tumors stained strongly positive for CLDN6, while one sample of osteosarcoma stained 50% strongly positive.

Due to the high prevalence of CLDN6 positivity in GCTs, the GCT subcohort was subjected to further analysis. Clinically, GCTs are a heterogeneous group of neoplasms that occur at various sites, both in the gonads and less commonly in extragonadal sites, and in both male and female patients from infancy to adulthood. In our study, a total of 29 GCT cases were analyzed for CLDN6 expression, including gonadal GCTs (GCTs of the testis (n=7), GCTs of the ovary (n=6)) and extragonadal GCTs (GCTs of the CNS/brain (intracranial, n=8), of the mediastinum (n=4), sacrococcygeal (n=3), and retroperitoneal (n=1)). Patient age ranged from 0 to 20 years (n=28 of 29 patients were ≤18 years) (see online supplemental table 2 for details). High CLDN6 expression, defined as medium (2+) or strongly positive (3+) CLDN6 expression in at least 50% of tumor cells, was detected in 20/29 cases (figure 3D). High CLDN6 expression was detected in all seminoma/germinoma/dysgerminoma cases (7/7), in all mixed germ cell tumors without an extensive teratoma component (9/9), as well as in pure embryonal carcinoma cases (2/2), one yolk sac tumor (1/1), and one choriocarcinoma (1/1). 9 of 29 GCT cases showed low CLDN6 expression ranging from 0% to 16% of moderately (2+) to strongly (3+) CLDN6 positive tumor cells. This low positive GCT subcohort included all five pure teratomas (including mature and immature teratoma cases) and four mixed germ cell tumors with an extensive teratoma component (>90% of tumor tissue).

To further understand the landscape of CLDN6 expression in pediatric tumors, a second tumor cohort was analyzed, which included hematolymphoid tumors (6 samples from 5 cancer types) and solid tumors (49 samples from 22 tumor entities) (online supplemental Table 3). The analysis of the second cohort supported the observation of high CLDN6 expression in non-teratoma GCTs and nephroblastoma. However, no CLDN6 expression was identified in cases of Ewing sarcoma/other embryonal tumors and hepatoblastoma. Similarly, no CLDN6 expression was identified in any of the investigated hematolymphoid tumors.

The above findings are supported by an analysis of CLDN6 transcript expression across a cohort of 2,518 pediatric cancers available on the OpenPBTA^32^ and OpenPedCan^33^ platforms. The highest expression was seen in the AT/RT-tyrosinase (TYR) subgroup (n=13, mean log transcripts per million (LogTPM) 4.919), followed by nephroblastomas (n=130, mean LogTPM=2.785), the AT/RT-MYC subgroup (n=19, mean LogTPM=2.733), intracranial GCTs (n=22, mean LogTPM=2.656), extracranial rhabdoid tumors (n=64, mean LogTPM=1.97), and the AT/RT-Sonic Hedgehog (SHH) subgroup (n=23, mean LogTPM=1.318) (online supplemental figure 1). The mean LogTPM for all AT/RTs was 2.658 (n=55). Lower CLDN6 transcripts were found in other tumor entities, with outlier elevated values noted for chordoma, high-grade glioma, ependymoma, medulloblastoma, neuroblastoma, and acute myeloid leukemia. Extracranial GCTs were not included in the analysis due to the small sample size (n=1) with RNA sequencing data available in OpenPedCan.

In summary, our data reveals that several solid pediatric tumor entities, including GCTs, nephroblastoma, and AT/RTs, strongly express CLDN6 in the majority of their cancer cells.

### CLDN6-CAR T cells selectively kill patient-derived CLDN6-expressing AT/RT cell lines in vitro

**Figure 5:**
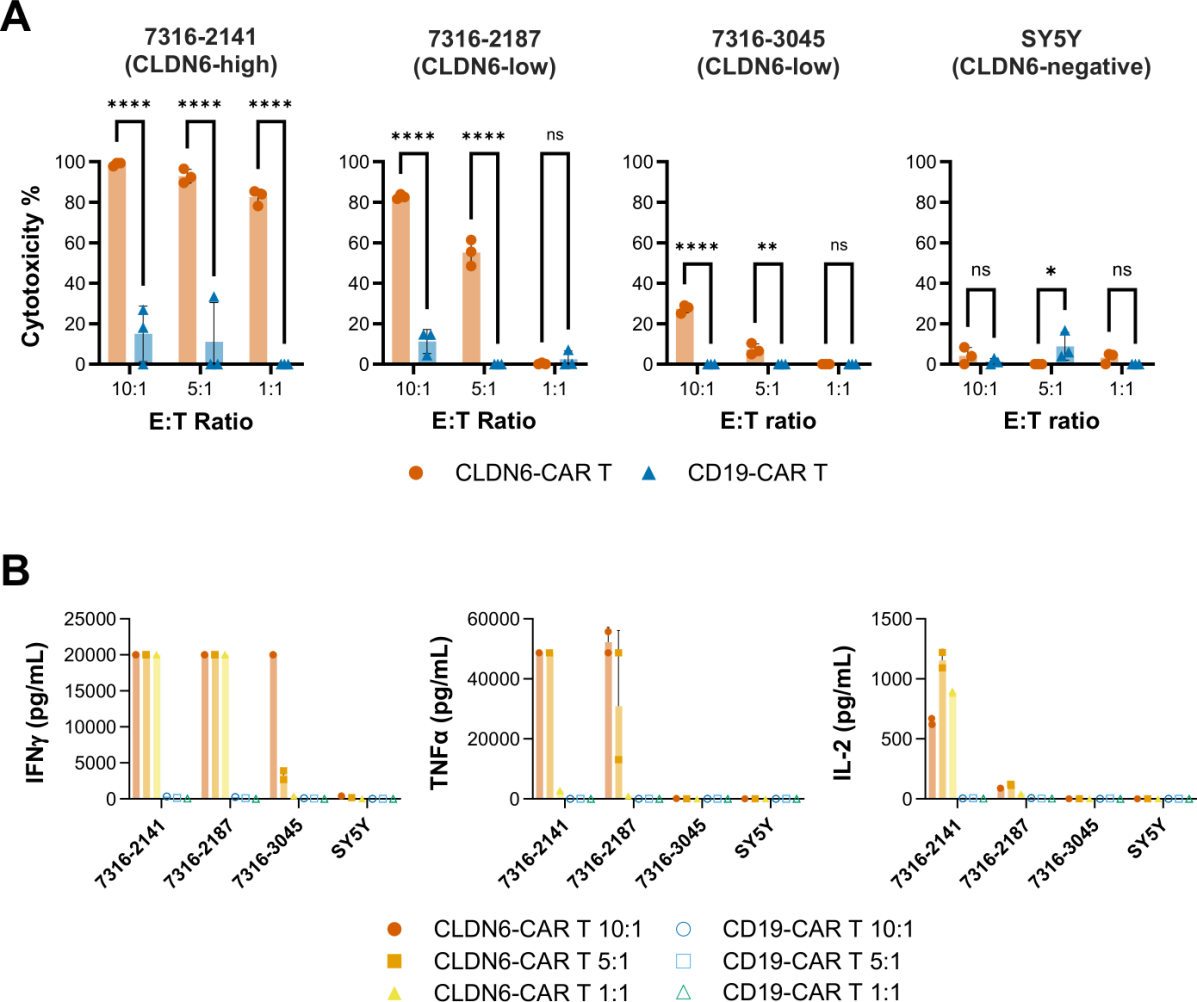
CLDN6-CAR T cells kill CLDN6-positive AT/RT cell lines and secrete effector cytokines in vitro. RNA-electroporated CLDN6-CAR T cells were co-cultured with high and low CLDN6-expressing AT/RT cell lines for 24 hours at different effector-to-target (E:T) ratios. CD19-CAR T cells and a CLDN6-negative cell line, SY5Y, were used as controls. (A) Specific target cell lysis, as evaluated by a luciferase-based cytotoxicity assay. (B) Cytokine concentrations in cell culture supernatants, as analyzed by cytokine multiplex assay after 48 hours co-culture. Bars and whiskers depict the means of triplicates ± the SD. Significance was assessed by two-way analysis of variance with Šídák’s multiple comparisons test: ****p<0.0001; **p<0.01; *p<0.05. AT/RT, atypical teratoid/rhabdoid tumor; CLDN6, claudin 6; IFN, interferon; IL, interleukin; TNF, tumor necrosis factor.

To confirm the susceptibility of CLDN6-expressing pediatric tumors to targeted therapies, we next assessed the in vitro cytotoxicity of second-generation CLDN6-CAR T cells, generated by RNA electroporation of the CAR, against three pediatric patient-derived AT/RT cell lines endogenously expressing high (7316–2141) or low (7316–2187, 7316–3045) levels of CLDN6 (figure 5). CLDN6 expression in these cell lines was assessed by flow cytometry (online supplemental figure 2 and 3). Quantification of CLDN6 molecules per cell revealed that CLDN6 expression in cell line 7316–2141 was approximately sevenfold higher than that in cell lines 7316–2187 or 7316–3045 (online supplemental figure 3). 1 day after transfection, CAR T cells showed high CAR positivity on the cell surface, which decayed over the course of 1 week (online supplemental figure 4), as previously described for RNA CAR T cells.^34^ CAR T cells targeting an irrelevant antigen (CD19) and a CLDN6-negative target cell line, the neuroblastoma cell line SY5Y, were used as controls. CLDN6-CAR T cells showed cytotoxicity against the high CLDN6-expressing AT/RT cell line at all effector-to-target (E:T) ratios, increasing concordantly with the effector dose, whereas cytotoxicity by CD19-CAR T cells was minimal (figure 5A). The low-CLDN6-expressing cell lines, 7316–2187 and 7316–3045, were also lysed by CLDN6-CAR T cells, but to a lesser extent than the high-CLDN6-expressing 7316–2141 cell line, and only at the higher E:T ratios of 10:1 and 5:1. No CAR T cell-mediated cytotoxicity was detected against the CLDN6-negative SY5Y cell line. Cytokine release in co-culture supernatants correlated with cytotoxicity, as evidenced by elevated levels of interferon-γ, tumor necrosis factor-α, and interleukin-2 (figure 5B). Additionally, real-time cell monitoring over several days revealed that CLDN6-CAR T cells suppressed the proliferation of low CLDN6-expressing 7316–3045 AT/RT cells compared with CD19-CAR T cells when co-cultured at E:T ratios of 1:1, 5:1, and 10:1 (online supplemental figure 5).

### CLDN6-CAR T cells mediate antitumor activity against orthotopic AT/RT xenografts in mice

**Figure 6:**
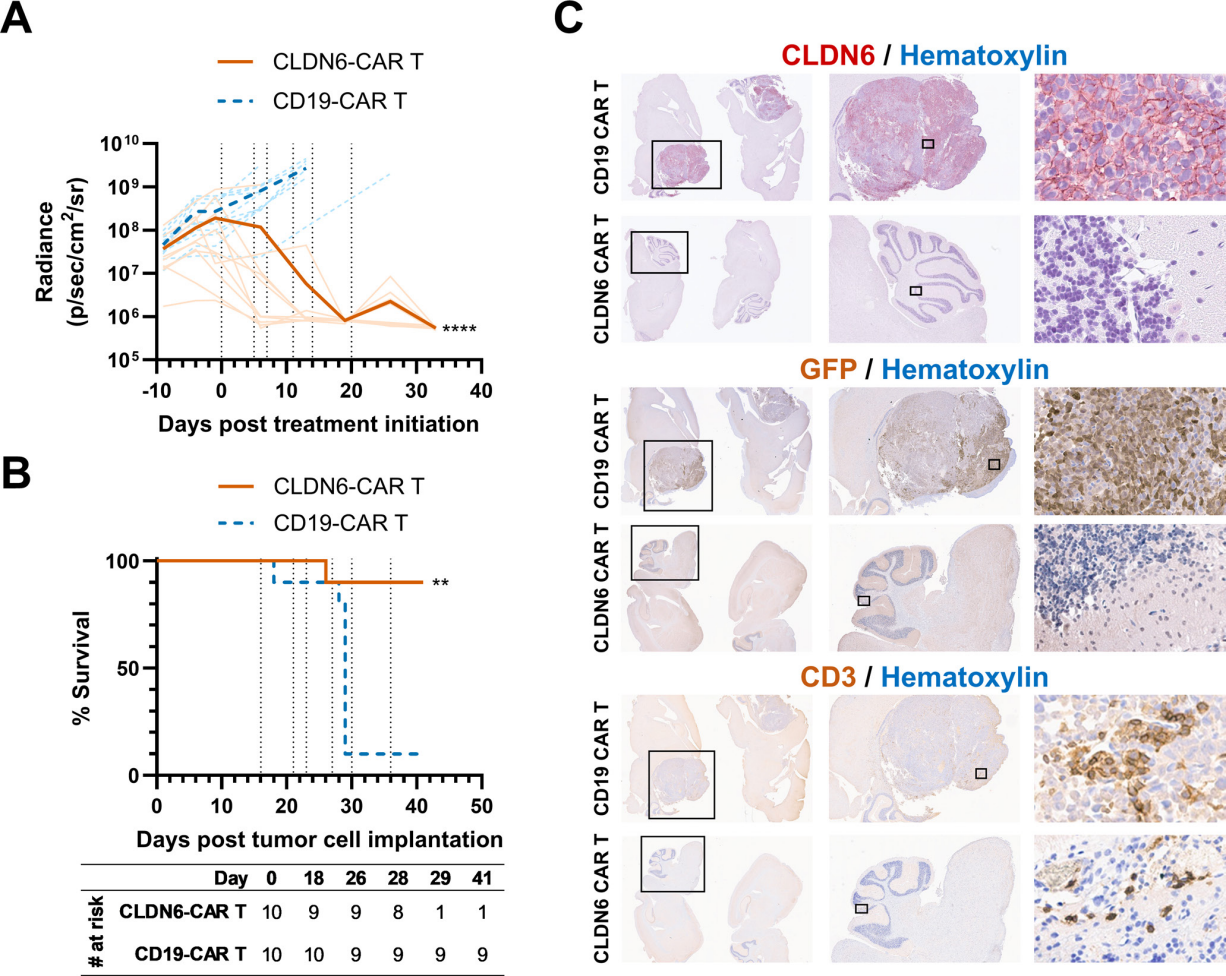
CLDN6-CAR T cells mediate tumor regression and prolong survival in orthotopic pediatric AT/RT mouse models. NSG mice (n=10/group) were implanted orthotopically in the brain with a patient-derived, endogenously CLDN6-expressing AT/RT cell line (7316–2141) engineered for constitutive GFP and firefly luciferase expression. Mice were then intratumorally inoculated with CLDN6-CAR or control CD19-CAR RNA-transfected T cells (5×10^6^ cells per dose) on days 16, 21, 23, 27, 30, and 36 after tumor implantation (vertical dotted lines). (A) Tumor burden expressed as mean radiance. Radiance curves were compared by mixed-effects analysis (****p<0.0001). Transparent lines represent individual mice. (B) Overall survival. Survival curves were compared by log-rank (Mantel-Cox) test (**p<0.01). (C) Representative IHC images of murine brains. IHC was performed on five mice per group with one representative staining shown. Specimens were collected at the time of death due to tumor progression for the CD19-CAR control group or at 33 days post-treatment initiation for the CLDN6-CAR group. CLDN6, H&E, and GFP staining were used for tumor identification, and CD3 staining was used to identify transferred T cells. In vivo experiments were performed three total times using two different T-cell donors in separate experiments with similar outcomes. The figure depicts one representative experiment. AT/RT, atypical teratoid/rhabdoid tumors; CAR, chimeric antigen receptor; CLDN6, claudin 6; GFP, green fluorescent protein; IHC, immunohistochemical; NSG, NOD-SCID-γc^–/–^.

We next evaluated susceptibility of endogenously CLDN6-expressing 7316–2141-derived AT/RTs to CAR T-cell therapy in vivo. NOD-SCID-γc–/– (NSG) mice bearing orthotopic intracranial AT/RTs containing green fluorescent protein (GFP) and luciferase engrafted to an average radiance of 1.25×10^8^ p/sec/cm^2^/sr. After guide cannulas were inserted into the tumor beds, animals were infused intratumorally with 5×10^6^ CLDN6-CAR or CD19-CAR RNA-transfected T cells two times a week for a total of six doses. Repeated dosing was employed due to the transient CAR expression by RNA-engineered CAR T cells, as previously described.^35^,^37^ Tumor burden was significantly reduced in mice treated with CLDN6-CAR T cells, while tumor growth continued in those treated with CD19-CAR T cells (figure 6a). The reduction in tumor burden translated into prolonged survival of the mice treated with CLDN6-CAR T cells compared with the control mice (p<0.01, figure 6b).

IHC analysis of brain tissues post-treatment showed a reduction in tumor size in the CLDN6-CAR T cell group; signals of CLDN6 and GFP (tumor) were low or absent in comparison to the control mice (figure 6c). T-cell infiltration was noted in inoculated brain areas of mice treated with the CLDN6-CAR T, with T cells also observed in control mice secondary to intratumoral administration. No toxicities, including neurologic toxicity, were observed in either group during treatment, and body weights remained stable (average weight 22.4 g for CLDN6-CAR group and 22.8 g for CD19-CAR group throughout study, p=not significant). The control mice developed neurologic symptoms that were secondary to large intracranial tumor burden as confirmed by bioluminescent imaging. Mice that survived beyond 40 days developed graft-versus-host disease from human T cells implanted in the mice across all groups, as we and others have previously observed.^36 38^ No toxicities were observed that could be related specifically to the CLDN6-CAR T-cell treatment.

## Conclusions/discussion

Over the past few decades, the incidence of pediatric cancer has been increasing, and cancer is now the second leading cause of death in children in the USA.^39 40^ Although treatment advancements have improved overall survival for many pediatric cancers, CNS tumors and metastatic/relapsed solid tumors continue to carry a dismal prognosis.^40^,^42^ For example, AT/RTs have a 5-year overall survival rate of under 50%, despite intense multimodal therapy,^5 6 43 44^ and once relapsed, it is incurable. As AT/RTs are the most common CNS tumors diagnosed in infants,^45^ these tumors are responsible for a significant number of life-years lost. To improve the outcomes for patients with these devastating tumors, novel approaches such as CAR T cell and other immunotherapies will be necessary, prompting our investigation into the therapeutic target CLDN6.

Taken together, our results reveal three key findings. First, semiquantitative IHC analysis of a large panel of normal pediatric tissues from infants to adolescents showed that CLDN6 cell-surface expression is absent in the vast majority of normal organs, including in those in which we found prenatal CLDN6 expression. Rare exceptions of positive CLDN6 staining in pediatric tissues included individual CLDN6-expressing cells in samples from the kidney, pancreas, pituitary, and salivary gland, which comprised less than 1% or 2% of the sample and with weakly positive CLDN6 staining (1+intensity). Our previous IHC analysis of 40 normal tissue types from adults found no CLDN6 expression in the tested organ panel.^23^ Our data suggest that this tight transcriptional repression of CLDN6 expression body-wide occurs at birth. This aligns with another study, which reported that CLDN6 expression was lost after birth, except for the first week of life.^27^ Taken together, these findings suggest that CLDN6-targeted therapies pose a low risk of on-target, off-tumor activity in children and adolescents.

Second, CLDN6 is frequently expressed in pediatric solid tumors with high medical need such as GCTs (except teratomas), nephroblastoma, extracranial MRTs, and AT/RTs. In these tumor entities, we observed CLDN6 cell-surface expression in substantial proportions of cells and most positive cells expressed CLDN6 with medium (2+) to strongly positive (3+) staining intensities. Moreover, substantial and strongly positive CLDN6 expression (50%–100% of cells staining 3+) was detected in isolated samples of hepatoblastoma, Ewing sarcoma/other embryonal tumors, osteosarcoma, and neuroblastoma. No CLDN6 expression was observed in a small cohort of pediatric hematolymphoid tumors of limited size with single cases per entity. Studies of larger sample sets are required to confirm this observation. A previous screening of pediatric tumor tissues for cell-surface CLDN6 expression found similar positivity rates in sample sets of GCTs, nephroblastoma, MRTs, and AT/RTs (>10% of cells were positive in 39%–100% of samples), though few GCTs were tested.^28^ Isolated positive samples were also identified in primitive neuroectodermal tumor (PNET), meningioma, and medulloblastoma, as well as strong expression observed in a single hepatoblastoma. Another IHC study found substantial CLDN6 expression in GCTs and one sample of desmoplastic small round cell tumor, as well as some expression in nephroblastomas.^27^ The present study’s large sample set and semiquantitative analysis add depth to the limited and conflicting knowledge on the landscape of CLDN6 cell-surface expression in pediatric tumor entities,^21 29^ and support CLDN6 as a potential biomarker and target for GCT, nephroblastoma, extracranial MRTs, and AT/RTs. Furthermore, our results and previous works suggest that CLDN6 may be a potent therapeutic target even in tumor entities where it is less frequently expressed, as expression in individual cases can be substantial and strongly positive.

Third, CLDN6-expressing pediatric AT/RTs are susceptible to a CAR T-cell therapeutic approach as shown against both primary tumor cell lines and mouse xenograft models. Both in vitro and in vivo data demonstrated potent target-dependent antitumor activity of CLDN6-CAR T cells. Because the CAR T cells were injected intratumorally, T-cell infiltration was noted in xenograft mice models treated with either CD19 or CLDN6-CAR T cells, but tumor burden was selectively reduced in those treated with CLDN6-CAR T cells. Intratumorally delivered CLDN6-CAR T cells also provided a significant survival advantage compared with control in xenograft models with no signs of off-target or off-tumor side effects. Furthermore, although late-onset graft-versus-host disease (GvHD) is known to occur in the NSG mouse model used, our data showed persistent tumor progression in mice receiving multiple administrations of control CAR T cells. This suggests that the observed therapeutic effect of CLDN6 CAR T cells is antigen-specific and indicates a relatively low likelihood of GvHD contributing to the early or primary phases of the antitumor response. In a phase 1 first-in-human clinical trial, CLDN6-CAR T cells have shown strong signals of clinical activity with a manageable safety profile in patients with CLDN6-positive solid tumors.^24^,^26^ Building on the recent and ongoing clinical trials using CAR T cells in children with solid tumors^10^ and delivered intracranially for pediatric CNS tumors,^9 11 46 47^ the present results support further investigation of the potential benefits of adoptive cell therapies in pediatric patients whose tumors express CLDN6.

This study has limitations. While a broad range of normal tissue types from four different age groups was analyzed, only one sample was analyzed per tissue type and age group. Tumor tissues included in the analysis were broad and included the vast majority of solid tumors encountered in pediatric patients; however, very rare solid tumors were not included. Follow-up studies of specific tumor types that are missing (eg, melanoma, carcinomas, thyroid and pancreatic tumors) or underpowered in this dataset (hematolymphoid tumors) would be required to fully assess CLDN6 expression across all pediatric tumors. Furthermore, although the solid tumor tissue microarrays (TMAs) were compiled by expert pathologists and neuropathologists in the field and represent the most accurate diagnoses at the time of compilation, they were constructed over multiple years. As such, the diagnostic criteria or terminology for some tumor entities may have changed during this time (eg, PNET), introducing potential diagnostic error. Additionally, CLDN6-CAR T cells were not tested against other CLDN6-positive tumor cell lines apart from AT/RTs. However, the potent and specific activity observed against AT/RTs is in line with previous observations for CLDN6-expressing adult tumors.^22^ Finally, in the in vivo experiments, the cell line used was derived from one child (<2 years of age) and experiments were performed in equal numbers of male and female mice. Donor T cells were not matched for sex. This combination could potentially introduce sex bias as a limitation of the experimental set-up.^23^

In summary, the CLDN6 expression profile in solid pediatric tissues, together with the observed efficacy of CLDN6-targeted CAR T cells against CLDN6-expressing solid tumors in experimental models, supports further studies of this modality as a potential novel therapy for hard-to-treat pediatric solid tumors, including those of the CNS.

## Methods

### RNA sequencing

RNA-sequencing analysis was performed for adult and pediatric samples publicly available through the OpenPedCan repository: https://github.com/d3b-center/OpenPedCan-analysis.^33^ Gene counts were filtered and normalized across tumors to TPM before being log-transformed for data visualization purposes. Analysis was completed using R V.4.3.1 (RStudio, Boston, Massachusetts, USA).

### Human histological samples

Slides of archived formalin-fixed, paraffin-embedded (FFPE) fetal tissues (miscarriages, fetal death or stillbirth) ranging from gestational age week 9–37.1 were provided by the tissue bank of the University Medical Center Mainz.

Representative tissue slides in TMAs of FFPE normal tissues were provided by the Children’s Hospital of Philadelphia (CHOP). 157 tissue samples from 42 tissue types were collected from a total of 78 donors across four age groups: 0–2, 2–8, 8–12.5 and 12.5–18 years. Specimens were collected from the archives of the anatomic pathology department at CHOP. Surgical samples were used whenever available or, when not available, autopsy cases were substituted with low post-mortem interval. All tissues were fixed in 10% neutral-buffered formalin and paraffin-embedded. Specimens were stored for up to 13 years prior to punching into the TMA. TMA blocks were stored at room temperature prior to microtome slicing onto slides.

TMAs of FFPE tissues from pediatric patients with solid tumors (527 tissue samples from 21 pediatric tumor entities) were obtained by CHOP in a similar manner to the normal tissue TMAs (dataset A). Archived solid and CNS tumors were selected by pathologists for inclusion into disease-specific TMAs based on histologic diagnoses. In total, nine tumor-specific CHOP TMAs were used in this study: two AT/RT arrays, neuroblastoma, medulloblastoma, neuroepithelial tumor, pediatric high-grade glioma, rhabdomyosarcoma, small round blue cell tumors, and other brain tumors.

An additional set of whole-slide FFPE tumor tissues from pediatric patients with hematolymphoid and solid tumors (49 tissue samples from 22 pediatric tumor entities) was provided by the Hannover Medical School (as a retrospective study of archived samples) and the University Medical Center of the Johannes Gutenberg University Mainz (as a prospective biomarker study) (dataset B). Clinical and demographic information was obtained by reviewing medical charts and pathology reports. Based on this information, all tumor cases were classified according to the most recent WHO Classification of Pediatric Tumors (fifth edition).

### Staining protocols and antibodies

IHC analyses and histological assessment of all tissue samples for CLDN6 expression were performed in the central histology laboratory at BioNTech SE, Mainz. Tissue slides were manually stained with a monoclonal mouse anti-human CLDN6 antibody (clone 58-4B-2; CLAUDENTIFY6, BioNTech Diagnostics, Mainz, Germany) and a negative control reagent according to the manufacturer’s instructions. Whole-slide scans of stained tissues were taken with a NanoZoomer s360 (Hamamatsu Photonics) using a 40× magnification. Picture processing was performed in NDP.view2.

### Histological assessment

All samples were analyzed by one (fetal and normal tissues) or two (tumor tissues) board-certified pathologists for CLDN6 expression. CLDN6 staining in neoplastic cells in tumor samples was evaluated using a semiquantitative score that takes into account both the intensity of staining and the percentage of stained neoplastic cells. Staining intensity was graded as negative (0), weakly positive (1+), moderately positive (2+), or strongly positive (3+), with only membranous staining considered positive. Values were reported as the average of both pathologists’ scores and duplicate samples (when available). Likewise, CLDN6 expression in all tissue types present (eg, epithelial cells, smooth muscle cells) was examined in fetal and pediatric normal tissue samples. The highest intensity of CLDN6 staining as well as the distribution pattern (single: <0–2% positive cells in the respective tissue type, focal: >2%–50% positive cells, and diffuse: >50% positive cells) was recorded, and the respective tissue types were described in the comment section. Embryonic rabbit kidney tissue served as a positive control for each IHC staining. CLDN6-positive samples were defined as samples with any tumor cells staining positive for CLDN6 at any intensity (1+, 2+, or 3+)

### Tumor cell lines

Patient-derived AT/RT cell lines were obtained from the Children’s Brain Tumor Network (CBTN). Multiomic data sets characterizing the cell lines are available at pedscbioportal.org.^3348^,^50^ Cell lines were cultured as per the CBTN specifications. AT/RT cell lines 7316–2141, 7316–2187, and 7316–4149 were cultured in suspension media with Dulbecco's modified eagle medium (DMEM)/nutrient mixture F12 base, 1% GlutaMax, 1% Pen-Strep, 2% B27, 1% N2, 0.02% epidermal growth factor (EGF), 0.02% fibroblast growth factor (FGF), and 0.025% heparin. AT/RT cell line 7316–3045 was cultured in adherent media with DMEM/F12 base, 1% Pen-Strep, 20% fetal bovine serum (FBS), 1% GlutaMax. Control cell line SH-SY5Y (neuroblastoma cell line, American Type Culture Collection [ATCC]) was cultured in DMEM/F12 base with 10% FBS. GFP and luciferase were introduced for constitutive expression using lentiviral plasmids according to manufacturer’s instructions (Cellomics Technology, PLV-10172–200).

CLDN6 expression was determined for each cell line by flow cytometry; cells were washed in fluorescence-activated cell sorting (FACS) buffer (500 mL phosphate-buffered saline (PBS), 10 mL FBS, 2 mL 0.5 M EDTA) and then incubated in human CLDN6 Alexa-Fluor-647-conjugated antibody (R&D Systems) at 1:400 for 30 min, in the dark, at 4°C. Mouse IgG2B Alexa-Fluor-647-conjugated antibody (R&D Systems) was used as an isotype control. Flow cytometry data were acquired on BD Accuri C6 (BD Biosciences) or FACSVerse (BD Biosciences) flow cytometers. Analysis was completed on FlowJo V.10.2 (TreeStar). Cells were gated for singlets prior to CLDN6 analysis. Antigen quantification was also performed using Quantibrite Beads (BD Biosciences) per manufacturer protocol.

### CAR T cells

For generation of the CAR constructs and RNA, DNA of a second-generation CAR containing a single-chain variable fragment (scFv) domain directed against either CLDN6 (derived from the IMAB206-C46S antibody) or CD19 (derived from FMC63 antibody) linked to CD3ζ and 4-1BB intracellular signaling domains was cloned into the 1658 vector plasmid (GenScript) for in vitro RNA production.^23^ The 1658 plasmid has been optimized for RNA generation and was the generous gift of Katalin Karikó. The plasmid includes a T7 promoter to drive transcription, as well as a Xenopus globin 3’-untranslated region (UTR) and tobacco etch virus 5’-UTR to enhance translation, and incorporated poly-A tail for stability.^34^ CAR plasmid DNA was linearized, and then RNA was synthesized using MEGAscript T7 RNA transcription kit and supplemented with m1Ψ triphosphate (TriLink) in place of uridine triphosphate (UTP). DsRNA was removed with RNase III digestion.^34^

For T-cell expansion and RNA electroporation, human T cells collected from de-identified healthy donors by the University of Pennsylvania Human Immunology Core were stimulated with CD3/CD28 microbeads (Gibco) at a ratio of 3:1 for 7 days. Beads were magnetically removed and cells allowed to expand for an additional 5–10 days until the mean cell volume reached less than 400 fL, at which point they were cryogenically frozen in FBS with 10% dimethyl sulfoxide (DMSO). Stimulated T cells were thawed for 24 hours prior to use and allowed to rest, then electroporated with 1 µg of RNA per 1×10^6^ T cells using the ECM 830 Square Wave Electroporation System (BTX) with a setting of 500 V, 700–800 µs.^34^

CAR T cells were evaluated for surface CAR expression by flow cytometry using protein L (1:100; GenScript) as previously described.^51^ Flow cytometry data were acquired on BD Accuri C6 (BD Biosciences) or FACSVerse (BD Biosciences) flow cytometers. Analysis was completed on FlowJo V.10.2 (TreeStar). Cells were gated on lymphocytes prior to evaluation of protein L.

### Cytotoxicity assays and cytokine release quantification

AT/RT cell lines were engineered for constitutive expression of firefly luciferase using lentiviral plasmid according to manufacturer’s instructions (Cellomics Technology, PLV-10172–200). CAR T cells were co-cultured with CLDN6-expressing AT/RT cell lines at 10:1, 5:1, and 1:1 E:T ratios in tumor cell medium. For suspension cell lines, luciferase activity was assessed using BrightGlo assay (Promega) following 48 hours of co-incubation.^34^ Tumor-cell death was calculated in comparison to AT/RT cells treated with triton detergent, which was set at 100% cytotoxicity. For adherent cell lines, cell proliferation was measured every hour using the real-time, impedance-based, xCELLigence System (RT-CES; F Hoffman La-Roche). 1×10^4^ cells were plated in adherent tumor cell line media and target or control CAR-transfected T cells were added at 24 hours. Cell index impedance was measured every hour according to instructions of the supplier. Cytokine levels were quantified from the supernatant of the cytotoxicity assays in a multiplex fashion by Eve Technologies (Calgary, Canada) according to their technical specifications. Means of duplicates as well as SD were calculated.

### Mouse studies

Male and female immunodeficient NOD-SCID-γc–/– (NSG) mice were obtained from Jax Laboratories or bred in-house under specific pathogen-free conditions and were housed and used at CHOP.

For tumor xenograft experiments, tumor cells with constitutive GFP and firefly luciferase expression were injected into the cerebellum of 6–10 weeks old mice as previously described.^36^ No inclusion/exclusion criteria were used. Following verification of engraftment by bioluminescence imaging, mice underwent cannulation surgery with placement of guide cannulas into the tumor bed.^52^ Mice were then stratified by a blinded technician between control and treatment groups to achieve even sex distribution and a normal distribution of tumor burden as measured by bioluminescence imaging, and 16 days after tumor implantation, 5×10^6^ CAR RNA-transfected T cells in 4–5 µL PBS were infused intratumorally two times a week for a total of six doses. Repeated dosing was employed due to the transient CAR expression by RNA-engineered CAR T cells, as has been previously described.^35^,^37^ Control mice received CD19-CAR T cells, and treatment mice received CLDN6-CAR T cells. Treatments were completed in random cage order to avoid bias. All animals were imaged weekly using an in vivo bioluminescence imaging system following intraperitoneal injection of luciferin. Measurements were also obtained in random cage order to avoid bias. The experimental plan aimed for 10 mice per group to power survival analysis. No animals were excluded from analyses. In vivo experiments were performed in triplicate. Two T-cell donors were used for CAR T-cell generation in separate experiments.

Outcome measures were tumor burden and survival. Tumor burden was extrapolated from mean radiance measurements. At the study endpoint (day 33 post start of treatment, or 49 days after tumor injection), mice were perfused with paraformaldehyde and the brains were harvested for IHC analysis.

The researchers were not blinded throughout the experiment.

### Histological analysis of mouse samples

Xenograft tumor samples from the in vivo mouse studies were analyzed by IHC staining for CLDN6, CD3, and GFP. CLDN6 staining was performed manually on FFPE brain sections using a monoclonal CLDN6 antibody (Cell Signaling; Cat. No. 18 932T). Tissue sections were subjected to heat-induced antigen retrieval for 10 min at 120°C in citrate buffer pH6 (Santa Cruz, Cat No. C999-100 mL), quenching of endogenous peroxidases by 0.3% H_2_O_2_ and blocking with 10% goat serum before incubation with the CLDN6 antibody (1:150) overnight at 2–8°C. After washing, slides were incubated with horseradish peroxidase (HRP)-coupled goat anti-rabbit secondary antibody (Immunologic, Cat. No. DPVR110HRP, ready to use). Signal development was carried out using Vector NovaRed (Vector Laboratories, SK-4800) for 4:30 min. Counterstaining was done using Richard-Allan-Scientific Mayer’s Hematoxylin (Therm, Cat. No. 72804) for 4 min. Whole-slide scans of stained tissues were taken with a NanoZoomer s360 (Hamamatsu Photonics) using a 40× magnification. Picture processing was performed in NDP.view2.

FFPE tissue slides were stained with CD3 antibody (Dako A0452) using the Bond-Max automated staining system (Leica Biosystems) and the Bond Refine Polymer Staining Kit (Leica Biosystems, DS9800). The standard protocol was followed, except for the primary antibody incubation, which was extended to 1 hour at room temperature. CD3 antibody was used at a 1:100 dilution. Antigen retrieval was performed using E1 (Leica Biosystems) retrieval solution for 20 min. After staining, the slides were rinsed, dehydrated through a series of ascending concentrations of ethanol and xylene, and coverslipped. The stained slides were then digitally scanned at 20× magnification using an Aperio CS-O slide scanner (Leica Biosystems).

FFPE tissue slides were stained with GFP (Invitrogen A11122). Antigen retrieval was done in a pressure cooker (Biocare Medical DC2012 “Decloaking Chamber”), set at 110°C for 15 min. The slides were rinsed two times in xylene for 5 min each and rehydrated in a series of descending concentrations of ethanol. Unmasking solution (Vector Labs H3300) was used to treat the slides in a pressure cooker. After cooling, the slides were rinsed in 0.1 M Tris Buffer and blocked with 2% fetal bovine serum for 5 min. The slides were then incubated with GFP antibody at a 1:100 dilution overnight at 4°. Following this, the slides were rinsed and incubated with biotinylated anti-Rabbit IgG (Vector Laboratories BA-1000) for 30 min at room temperature. The avidin-biotin complex (Vector Laboratories PK-6100) was then added to the slides and incubated for 30 min at room temperature. The slides were then rinsed and incubated with DAB (DAKO Cytomation K3468) for 10 min at room temperature. Finally, the slides were counterstained with hematoxylin, rinsed, dehydrated through a series of ascending concentrations of ethanol and xylene, and coverslipped. The dried slides were scanned at 20× magnification using an Aperio CS-O (Leica Biosystems) slide scanner.

### Statistical analyses

Statistical assessments were performed using GraphPad Prism software V.9 (GraphPad Software, La Jolla, California, USA). Means were compared using analysis of variance or Student’s t-test and displayed with SD. Overall survival was calculated using Kaplan-Meier curves with log-rank test.

## Supplementary material

10.1136/jitc-2025-011709 online supplemental file 1

## Data Availability

All data relevant to the study are included in the article or uploaded as supplementary information.
