# Supplementary material for: Claudin 6 is a suitable target for CAR T-cell therapy in atypical teratoid/rhabdoid brain tumors and other pediatric solid tumors
Source: J Immunother Cancer. 2025 Oct 10;13(10):e011709. doi: 10.1136/jitc-2025-011709 (PMC12516980; doi:10.1136/jitc-2025-011709)
Supplement: online supplemental file 1 [file jitc-13-10-s001.docx]

**Supplemental information for:**

CLAUDIN 6 IS A SUITABLE TARGET FOR CAR T CELL THERAPY IN ATYPICAL TERATOID/RHABDOID BRAIN TUMORS AND OTHER PEDIATRIC SOLID TUMORS

Peter J. Madsen^1,2,3,†^, Anna Melissa Schlitter^4,5,†^, Carina Flemmig^4,†^, Conor Dickson^3^, Kyra Harvey^6^, Cullen Wilson^3^, Ezra Beaubien^6^, Luke Patterson^6^, Allison Stern^3^, Crystal Griffin^6^, Nikhil Joshi^6^, Sreehita Hajeebu^6^, Daniel Martinez^7^, Phillip B. Storm^1,2,3^, Adam C. Resnick^1,2,3^, Peter Hillemanns^8^, Martin Stanulla^9^, Jörg Faber^10,11^, Arthur Wingerter^10,11^, Matthias Martin Gaida^12,13,14^, Saskia Holtemeyer^4^, Mark Laible^4^, Anja Feldner^4^, Florian Frohns^4^, João H. Duarte^15^, Bruno Valentin Sinn^4^, Stefan Wöll^4^, Ugur Sahin^4,11,16^, Özlem Türeci^4,16,‡,*^, Jessica B. Foster^6,17,‡,*^

**Affiliations**:

1. Division of Neurosurgery, Children's Hospital of Philadelphia, Philadelphia, PA, USA
2. Department of Neurosurgery, Perelman School of Medicine, University of Pennsylvania, Philadelphia, PA, USA
3. Center for Data Driven Discovery in Biomedicine (D3b), Children’s Hospital of Philadelphia, Philadelphia, PA, USA
4. BioNTech SE, Mainz, Germany
5. TUM School of Medicine and Health, Munich, Germany
6. Division of Oncology and Center for Childhood Cancer Research, Children's Hospital of Philadelphia, Philadelphia, PA, USA
7. Pathology and Laboratory Medicine, Children's Hospital of Philadelphia and Perelman School of Medicine at the University of Pennsylvania, Philadelphia, PA, USA
8. Departments of Gynecology and Obstetrics, and Comprehensive Cancer Center, Hannover Medical School, Hannover, Germany
9. Department of Paediatric Haematology and Oncology, Hannover Medical School, Hannover, Germany
10. Department of Pediatric Hematology/Oncology/Hemostaseology, Center for Pediatric and Adolescent Medicine, University Medical Center of the Johannes Gutenberg University Mainz, Mainz, Germany
11. University Cancer Center Mainz (UCT Mainz), University Medical Center of the Johannes Gutenberg-University of Mainz, Germany
12. Institute of Pathology, University Medical Center of the Johannes Gutenberg University Mainz, Mainz, Germany
13. TRON, Translational Oncology at the University Medical Center of the Johannes Gutenberg University Mainz, Mainz, Germany
14. Research Center for Immunotherapy, University Medical Center of the Johannes Gutenberg University Mainz, Mainz, Germany
15. BioNTech Cell & Gene Therapies GmbH, Mainz, Germany
16. HI-TRON (Helmholtz Institute for Translational Oncology) Mainz by DKFZ, Mainz, Germany
17. Department of Pediatrics, Perelman School of Medicine at the University of Pennsylvania; Philadelphia, PA, USA

^†^ Equal contribution

^‡^ Equal contribution

* Corresponding authors: Jessica B. Foster ([FOSTERJB@chop.edu](mailto:FOSTERJB@chop.edu)), Özlem Türeci ([Oezlem.Tuereci@biontech.de](mailto:Oezlem.Tuereci@biontech.de))

## Supplemental table 1

Supplemental Table 1. CLDN6 expression in solid pediatric tumors

The frequency of CLDN6 protein expression in 527 pediatric tumor tissue samples from 21 tumor entities (dataset A, see Methods) was determined using a semiquantitative immunohistochemistry (IHC) assay. Specimens from one or more individuals (N) were assessed per tumor entity. Two board-certified pathologists evaluated each sample and determined the proportion of cells staining negative (0), weakly positive (1+), medium positive (2+), or strongly positive (3+) for CLDN6. The mean and standard deviation of the proportion of CLDN6-positive cells per tumor entity are given for patient samples with any CLDN6 positivity (n).

|  | | **Positive samples^a^** | |  | **Mean proportion positive tumor cells in positive samples by staining intensity** | | | | | | | |
| --- | --- | --- | --- | --- | --- | --- | --- | --- | --- | --- | --- | --- |
|  |  |  |  |  | **1+/2+/3+** | | **3+** | | **2+** | | **1+** | |
| **Tumor entity** | **N** | **n** | **%** |  | **%** | **SD** | **%** | **SD** | **%** | **SD** | **%** | **SD** |
| **GCTs^b^** | **29** | 27 | 93.1 |  | 73.7 | 39.4 | 69.12 | 41.4 | 3.0 | 7.1 | 1.6 | 5.8 |
| **CNS tumors (excluding GCTs)** | **361** |  |  |  |  |  |  |  |  |  |  |  |
| AT/RTs | 51 | 20 | 39.2 |  | 34.1 | 26.9 | 20.1 | 27.2 | 8.4 | 7.0 | 5.6 | 9.2 |
| Choroid plexus papilloma | 11 | 0 | – |  | – | – | – | – | – | – | – | – |
| Craniopharyngioma | 17 | 0 | – |  | – | – | – | – | – | – | – | – |
| Ependymal tumors | 1 | 0 | – |  | – | – | – | – | – | – | – | – |
| Glioma, high-grade | 87 | 0 | – |  | – | – | – | – | – | – | – | – |
| Glioma, low-grade | 11 | 0 | – |  | – | – | – | – | – | – | – | – |
| Glioneuronal tumors | 32 | 0 | – |  | – | – | – | – | – | – | – | – |
| Medulloblastoma | 65 | 0 | – |  | ­– | – | – | – | – | – | – | – |
| Meningioma | 9 | 0 | – |  | – | – | – | – | – | – | – | – |
| Neuroblastoma | 72 | 0 | – |  | – | – | – | – | – | – | – | – |
| Oligodendroglioma | 5 | 0 | – |  | – | – | – | – | – | – | – | – |
| **Soft tissue tumors** | **74** |  |  |  |  |  |  |  |  |  |  |  |
| Extracranial MRTs | 16 | 8 | 50.0 |  | 38.4 | 34.5 | 27.3 | 31.5 | 8.5 | 13.2 | 2.6 | 4.4 |
| Ewing sarcoma and other embryonal tumors | 9 | 1 | 11.1 |  | 100 | – | 100 | – | 0 | – | 0 | – |
| Rhabdomyosarcoma | 41 | 0 | – |  | – | – | – | – | – | – | – | – |
| Synovial Sarcoma | 7 | 0 | – |  | – | – | – | – | – | – | – | – |
| DSRCT | 1 | 0 | – |  | – | – | – | – | – | – | – | – |
| **Other** | **63** |  |  |  |  |  |  |  |  |  |  |  |
| Nephroblastoma | 14 | 9 | 64.3 |  | 34.2 | 28.4 | 20.9 | 25.4 | 9.3 | 12.7 | 3.9 | 4.0 |
| Hepatoblastoma | 7 | 1 | 14.3 |  | 100 | – | 100 | – | 0 | – | 0 | – |
| Osteosarcoma | 38 | 2 | 5.3 |  | 25.1 | 35.2 | 25 | 35.4 | – | – | 0.1 | 0.2 |
| Retinoblastoma | 4 | 0 | – |  | – | – | – | – | – | – | – | – |
| ^a^ Defined as samples with any tumor cells staining positive for CLDN6 at any intensity (1+, 2+, or 3+). ^b^ See Supplemental Table 3 for a detailed evaluation of the pediatric GCT samples. Abbreviations: AT/RT, atypical teratoid/rhabdoid tumor; CLDN6, Claudin-6; DSRCT, desmoplastic small round cell tumors, GCT, germ cell tumor; MRT, malignant rhabdoid tumor; SD, standard deviation. | | | | | | | | | | | | |

## Supplemental table 2

Supplemental Table 2: Clinicopathologic evaluation of pediatric germ cell tumors (GCTs) (Dataset A)

Detailed evaluation of CLDN6 expression in pediatric GCTs (n=29) considering gonadal/extragonadal appearance, primary site, age, and histological subtype.

|  |  |  | **% CLDN6-positive cells by staining intensity** | | | | |
| --- | --- | --- | --- | --- | --- | --- | --- |
| **Diagnosis** | **Histological subtype** | **Age (years)** | **1+/2+/3** | **2+/3+** | **3+** | **2+** | **1+** |
| **Gonadal GCTs (n=13)** |  |  |  |  |  |  |  |
| GCT of the testis (n=7) | | | | | | | |
| Testis | non-seminoma, embryonal carcinoma | 13-20 | 100 | 100 | 100 | 0 | 0 |
| Testis | non-seminoma, embryonal carcinoma | 13-20 | 100 | 100 | 100 | 0 | 0 |
| Testis | mixed GCT | 13-20 | 100 | 100 | 100 | 0 | 0 |
| Testis | mixed GCT | 13-20 | 100 | 100 | 100 | 0 | 0 |
| Testis | mixed GCT | 13-20 | 100 | 100 | 100 | 0 | 0 |
| Testis | mixed GCT | 13-20 | 55 | 55 | 55 | 0 | 0 |
| Testis | seminoma/germinoma/dysgerminoma | 9-12 | 100 | 100 | 100 | 0 | 0 |
| GCT of the ovary (n=6) | | | | | | | |
| Ovary | mixed GCT | 9-12 | 87.5 | 57.5 | 30 | 27.5 | 30 |
| Ovary | mixed GCT | 9-12 | 72.5 | 70 | 45 | 25 | 2.5 |
| Ovary | mixed GCT with >90% teratoma component | 13-20 | 16 | 16 | 16 | 0 | 0 |
| Ovary | mixed GCT with >90% teratoma component | 9-12 | 10 | 10 | 9 | 1 | 0 |
| Ovary | seminoma/germinoma/dysgerminoma | 13-20 | 100 | 100 | 95 | 5 | 0 |
| Ovary | non-seminoma, yolk sac tumor | <2 | 100 | 100 | 100 | 0 | 0 |
| **Extragonadal GCTs (n=16)** | | | | | | | |
| Brain/CNS | mixed GCT | 9-12 | 100 | 100 | 100 | 0 | 0 |
| Brain/CNS | seminoma/germinoma/dysgerminoma | 13-20 | 100 | 100 | 100 | 0 | 0 |
| Brain/CNS | seminoma/germinoma/dysgerminoma | 13-20 | 100 | 100 | 100 | 0 | 0 |
| Brain/CNS | seminoma/germinoma/dysgerminoma | 13-20 | 100 | 100 | 100 | 0 | 0 |
| Brain/CNS | seminoma/germinoma/dysgerminoma | 9-12 | 100 | 100 | 100 | 0 | 0 |
| Brain/CNS | seminoma/germinoma/dysgerminoma | 13-20 | 100 | 100 | 100 | 0 | 0 |
| Brain/CNS | non-seminoma, teratoma | 13-20 | 0 | 0 | 0 | 0 | 0 |
| Brain/CNS | non-seminoma, teratoma | 9-12 | 0 | 0 | 0 | 0 | 0 |
| Mediastinum | mixed GCT with >90% teratoma component | 9-12 | 12.5 | 10 | 6.5 | 3.5 | 2.5 |
| Mediastinum | non-seminoma, teratoma | 13-20 | 11.25 | 7.25 | 4.25 | 3 | 4 |
| Mediastinum | non-seminoma, teratoma | 13-20 | 7.5 | 5 | 2.5 | 2.5 | 2.5 |
| Mediastinum | non-seminoma, teratoma | <2 | 1.5 | 1 | 0.5 | 0.5 | 0.5 |
| Sacrococcygeal region | mixed GCT | <2 | 100 | 100 | 90 | 10 | 0 |
| Sacrococcygeal region | mixed GCT | 2-8 | 100 | 100 | 100 | 0 | 0 |
| Sacrococcygeal region | mixed GCT with >90% teratoma component | <2 | 15 | 15 | 12.5 | 2.5 | 0 |
| Retroperitoneal | choriocarcinoma | 13-20 | 100 | 100 | 100 | 0 | 0 |
| Note: 28 of 29 patients were ≤18 years of age. Abbreviations: GCT, Germ cell tumor. | | | | | | | |

## Supplemental table 3

Supplemental Table 3. CLDN6 expression in pediatric hematolymphoid and other tumors

The frequency of CLDN6 protein expression in 49 pediatric tumor tissue samples from 22 tumor entities (dataset B, see Methods) was determined using a semiquantitative immunohistochemistry (IHC) assay. Specimens from one or more individuals (N) were assessed per tumor entity. Two board-certified pathologists evaluated each sample and determined the proportion of cells staining negative (0), weakly positive (1+), medium positive (2+), or strongly positive (3+) for CLDN6. The mean and standard deviation of the proportion of CLDN6-positive cells per tumor entity are given for patient samples with any CLDN6 positivity (n).

|  | | **Positive samples^a^** | |  | **Mean proportion positive tumor cells in positive samples by staining intensity** | | | | | | | |
| --- | --- | --- | --- | --- | --- | --- | --- | --- | --- | --- | --- | --- |
|  |  |  |  |  | **1+/2+/3+** | | **3+** | | **2+** | | **1+** | |
| **Tumor entity** | **N** | **n** | **%** |  | **%** | **SD** | **%** | **SD** | **%** | **SD** | **%** | **SD** |
| **GCTs** | **6** |  |  |  |  |  |  |  |  |  |  |  |
| Mixed GCT | 1 | 1 | 100 |  | 100 | – | 95 | – | 5 | – | – | – |
| Non-seminoma, teratoma | 5 | 0 | – |  | – | – | – | – | – | – | – | – |
| **CNS tumors (excluding GCTs)** | **10** |  |  |  |  |  |  |  |  |  |  |  |
| Neuroblastoma | 6 | 0 | – |  | – | – | – | – | – | – | – | – |
| Astrocytoma | 2 | 0 | – |  | – | – | – | – | – | – | – | – |
| Malign peripheral nerve sheath tumor | 1 | 0 | – |  | – | – | – | – | – | – | – | – |
| Paraganglioma | 1 | 0 | – |  | – | – | – | – | – | – | – | – |
| **Soft tissue tumors** | **9** |  |  |  |  |  |  |  |  |  |  |  |
| Angiosarcoma | 1 | 0 | – |  | – | – | – | – | – | – | – | – |
| Ewing sarcoma and other embryonal tumors | 6 | 0 | – |  | – | – | – | – | – | – | – | – |
| Fibrosarcoma | 1 | 0 | – |  | – | – | – | – | – | – | – | – |
| Rhabdomyosarcoma | 1 | 0 | – |  | – | – | – | – | – | – | – | – |
| **Other** | **18** |  |  |  |  |  |  |  |  |  |  |  |
| Nephroblastoma | 5 | 4 | 80 |  | 55.4 | 41.6 | 44.4 | 38.1 | 9 | 7.5 | 2 | 1 |
| Adenocarcinoma, not further specified | 1 | 0 | – |  | – | – | – | – | – | – | – | – |
| Congenital mesoblastic nephroma | 1 | 0 | – |  | – | – | – | – | – | – | – | – |
| Hepatoblastoma | 4 | 0 | – |  | – | – | – | – | – | – | – | – |
| Granulosa cell tumor | 1 | 0 | – |  | – | – | – | – | – | – | – | – |
| Osteosarcoma | 4 | 0 | – |  | – | – | – | – | – | – | – | – |
| Pleuropulmonary blastoma | 1 | 0 | – |  | – | – | – | – | – | – | – | – |
| Thymoma | 1 | 0 | – |  | – | – | – | – | – | – | – | – |
| **Hematolymphoid tumors** | **6** |  |  |  |  |  |  |  |  |  |  |  |
| Burkitt lymphoma | 2 | 0 | – |  | – | – | – | – | – | – | – | – |
| B-lymphoblastic leukemia / lymphoma | 1 | 0 | – |  | – | – | – | – | – | – | – | – |
| Diffuse large B-cell lymphoma | 1 | 0 | – |  | – | – | – | – | – | – | – | – |
| Hodgkin-lymphoma | 1 | 0 | – |  | – | – | – | – | – | – | – | – |
| Monomorphic B-cell PTLD | 1 | 0 | – |  | – | – | – | – | – | – | – | – |
| ^a^ Defined as samples with any tumor cells staining positive for CLDN6 at any intensity (1+, 2+, or 3+). Abbreviations: CNS, central nervous system; GCT, germ cell tumors; PTLD, post-transplant lymphoproliferative disorder | | | | | | | | | | | | |

## Supplemental figure 1


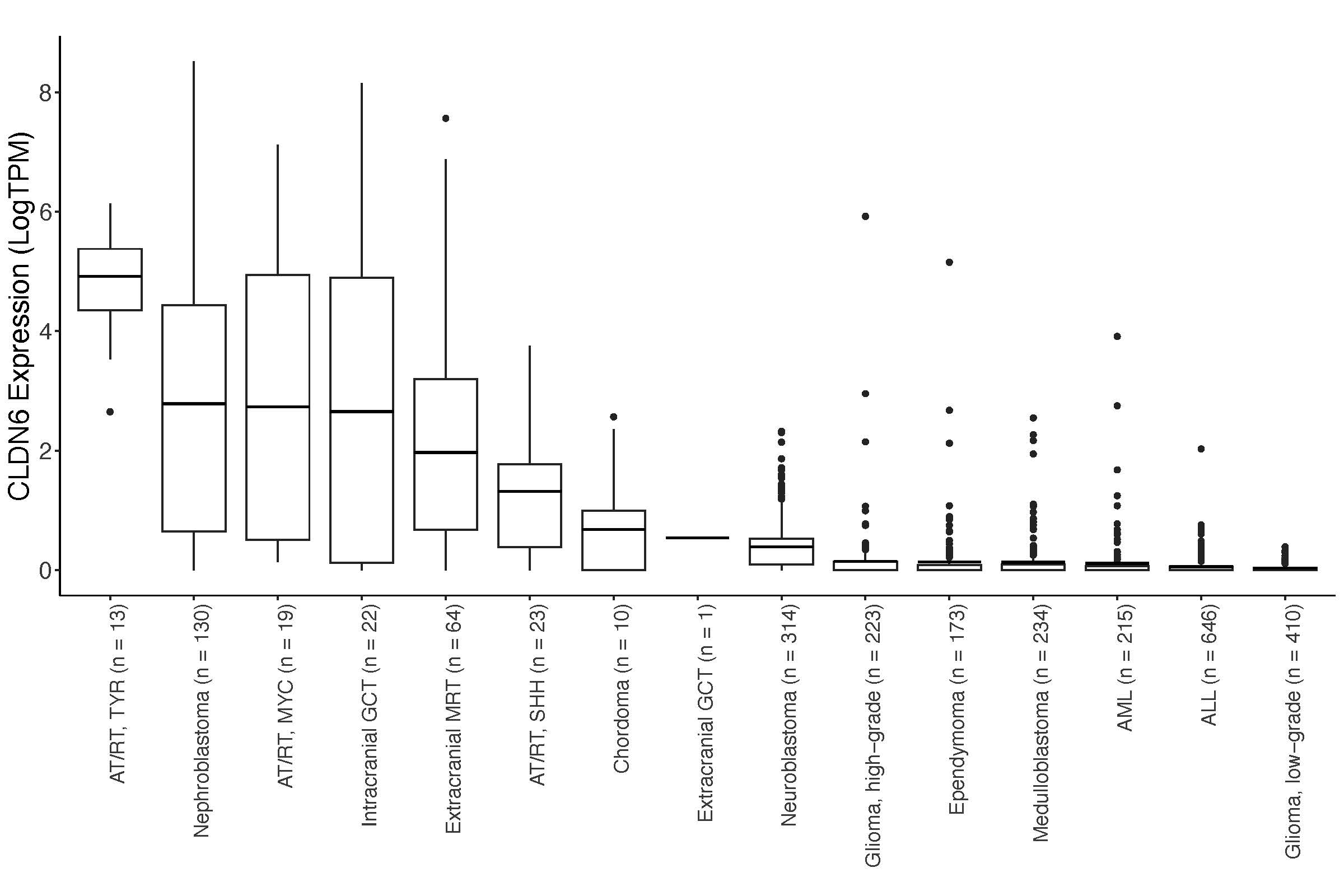


Supplemental Figure 1: CLDN6 RNA expression across pediatric cancers including AT/RT subtypes

Bulk RNAseq from OpenPedCan across 12 pediatric cancer types analyzed for expression levels of CLDN6. Data shown as mean indicated by the horizontal line, 25-75 percentile indicated by the box, +/- 1.5 interquartile range indicated by the vertical lines, and outlier samples indicated by dots. GCT = germ cell tumor; MRT = malignant rhabdoid tumor; AT/RT = atypical teratoid/rhabdoid tumor; HGG = high-grade glioma; NB = neuroblastoma; EPEN = ependymoma; AML = acute myeloid leukemia; MB = medulloblastoma; ALL = acute lymphoblastic leukemia/lymphoma; LGG = low grade glioma.

## Supplemental figure 2


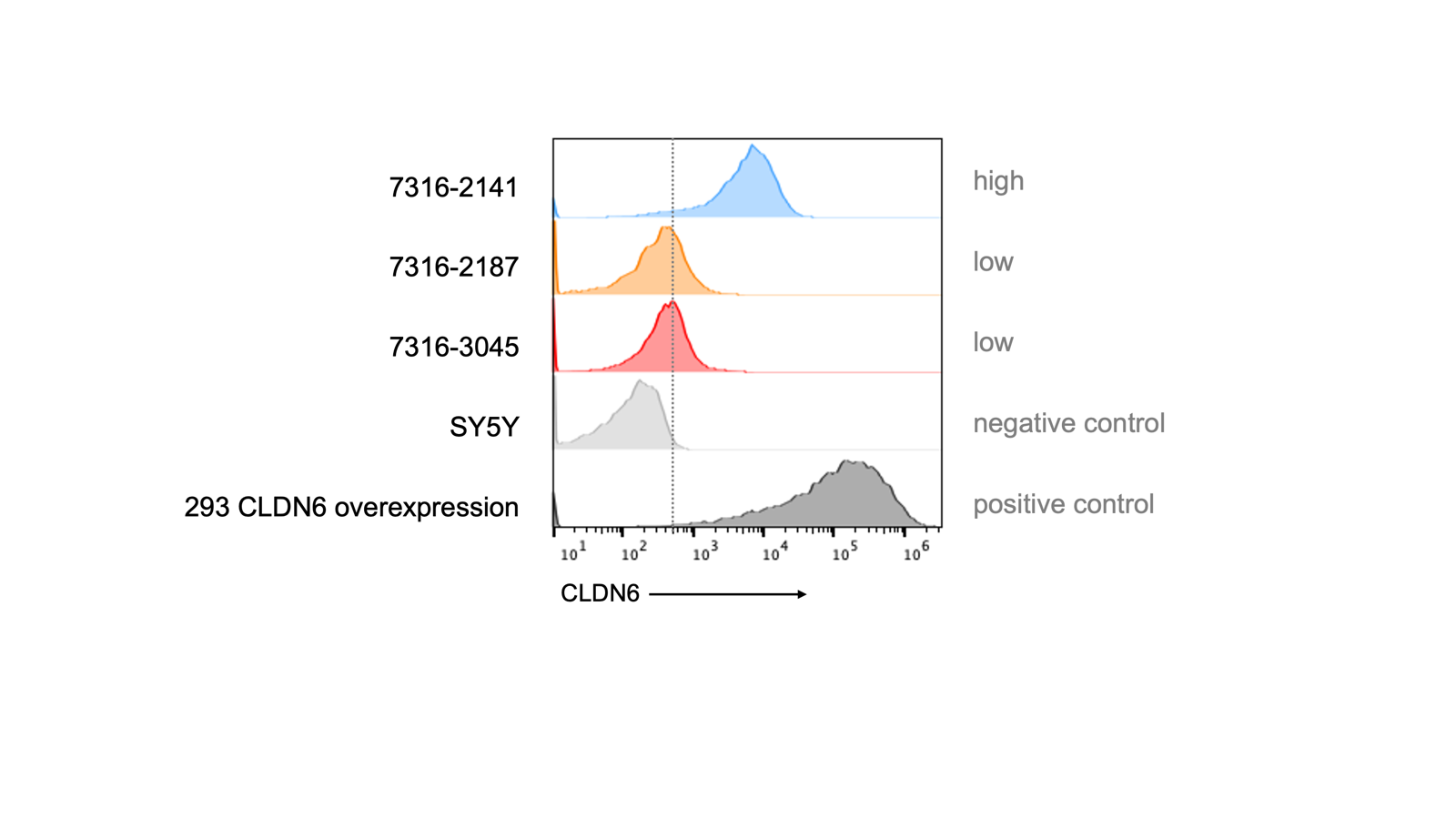


Supplemental Figure 2. CLDN6 expression in patient-derived AT/RT cell lines.

Four AT/RT cell lines show variable expression of CLDN6 within the range of negative and positive control CLDN6 staining. Flow cytometry histograms showing CLDN6 expression in AT/RT cell lines. Cell lines identified on the left with comparative level of expression on the right. Positive control (293T cells transfected to express CLDN6) and negative control (SY5Y) are shown at the bottom.

## Supplemental figure 3


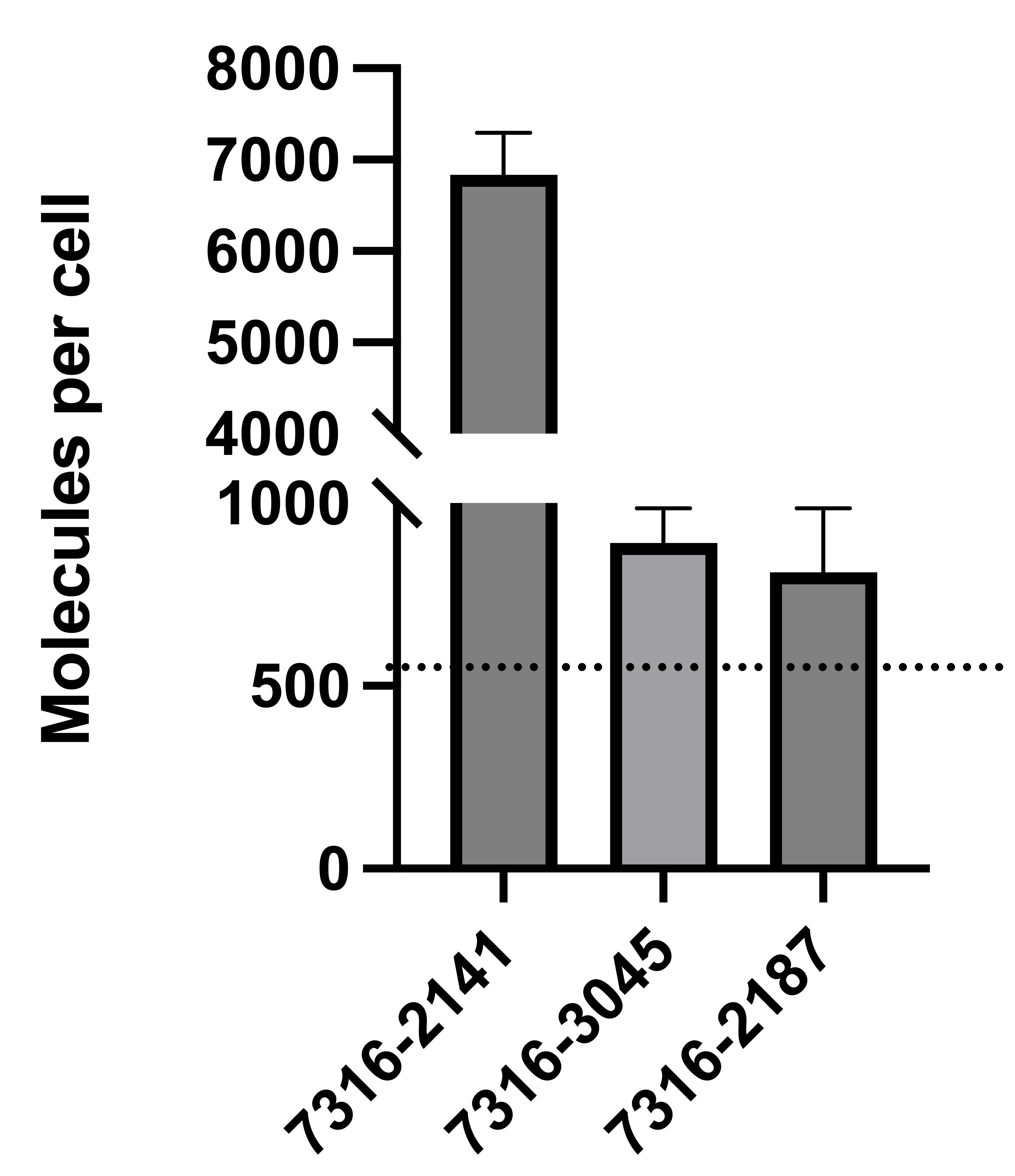


Supplemental Figure 3. Estimation of CLDN6 molecules per cell for four AT/RT cell lines.

Using a quantitative phycoerythrin bead assay, the amount of CLDN6 molecules on each cell was estimated. Untransfected HEK 293T cell line served as negative control and is represented by dotted line. Values displayed as mean + SD.

## Supplemental figure 4


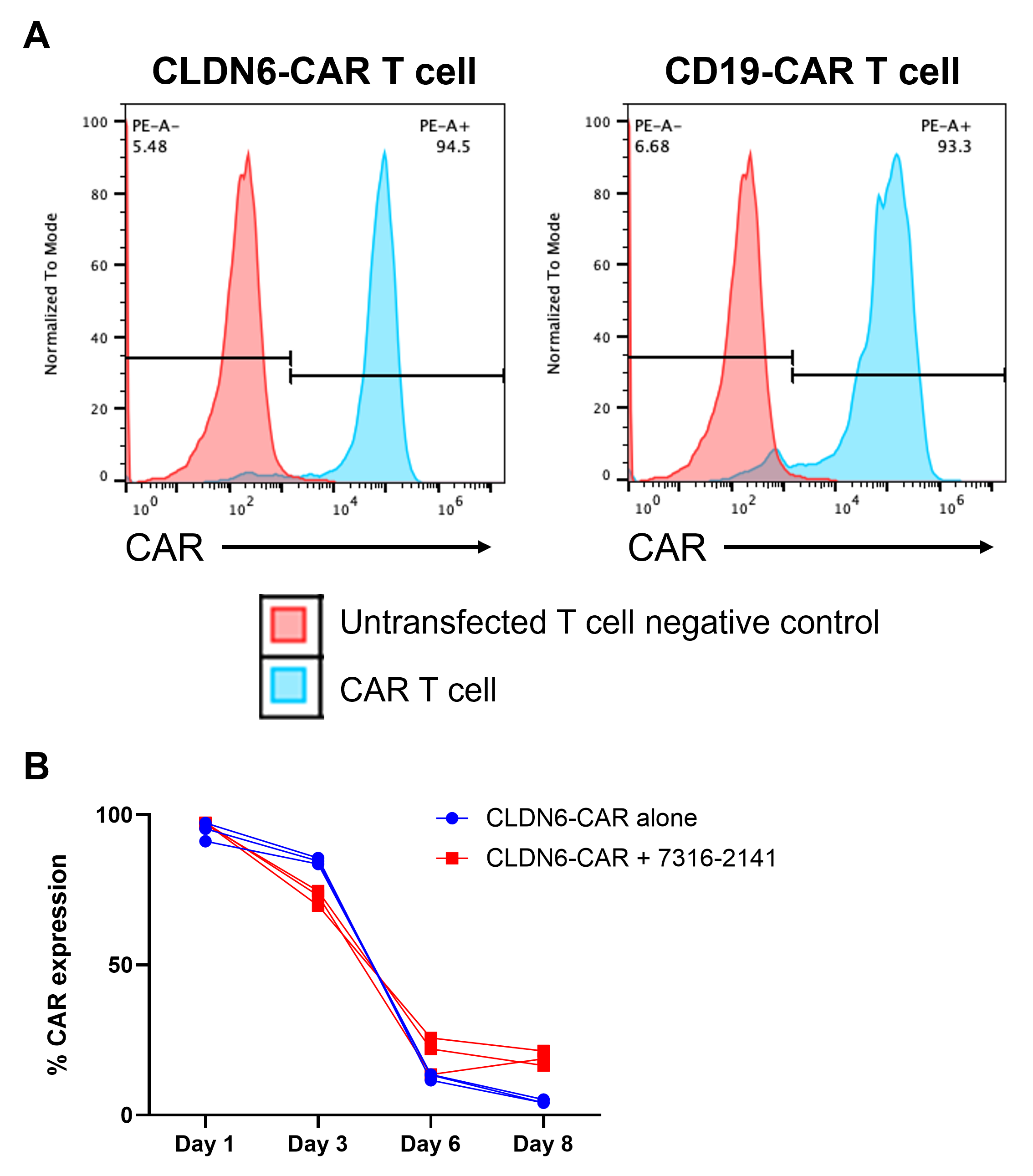


Supplemental Figure 4. CAR surface expression on T cells following RNA electroporation.

(A) Representative flow cytometry of CAR T cells stained with protein L to detect surface expression of CAR 24 hours following electroporation. (B) CLDN6-CAR expression determined by flow cytometry over time. The percentage of CAR-positive T cells was calculated for T cells in culture alone (blue line) or co-cultured with AT/RT cell line 7316-2141 (red).

## Supplemental figure 5


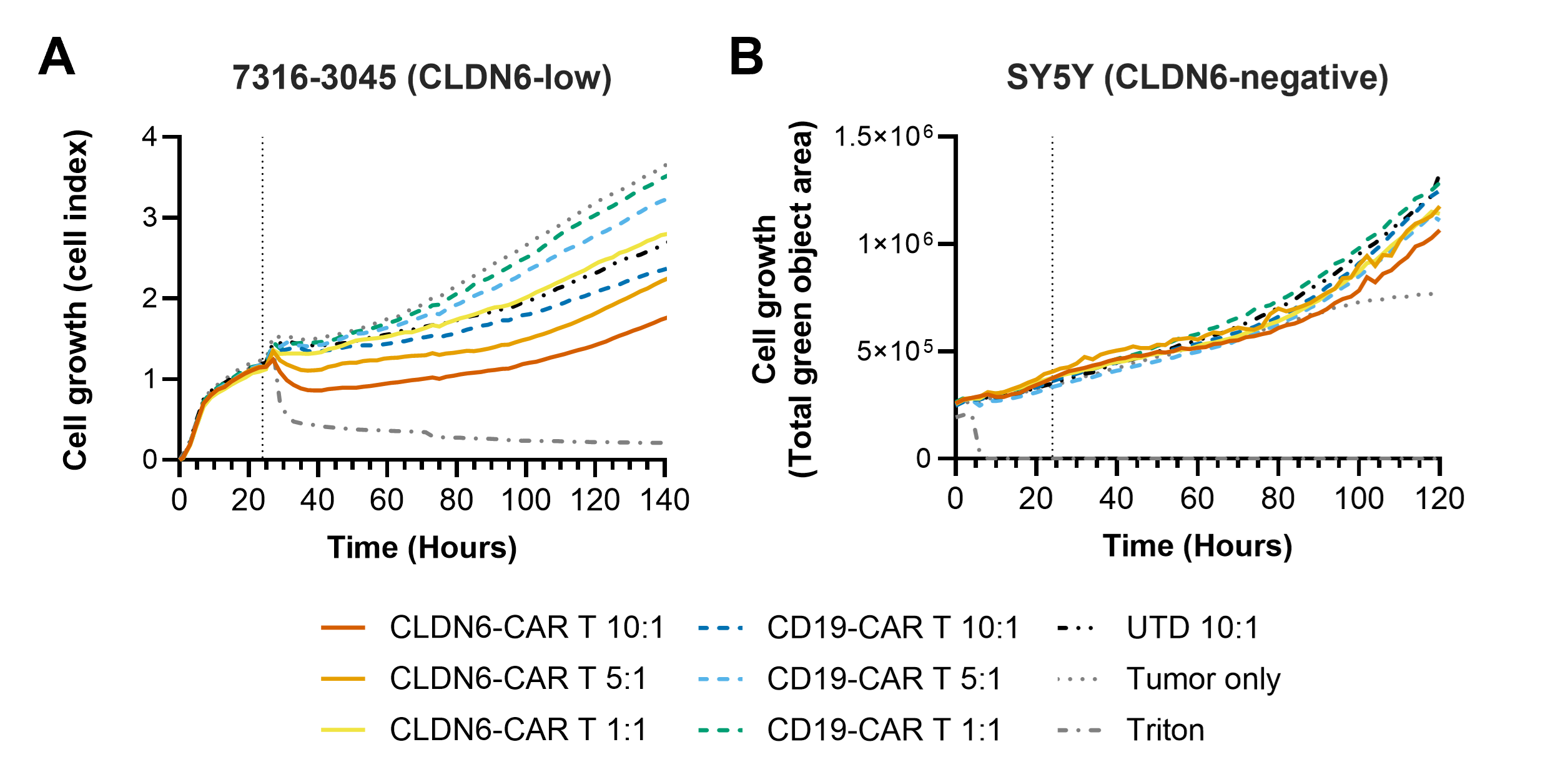


Supplemental Figure 5. CLDN6-CAR T cell-mediated growth inhibition of CLDN6-expressing AT/RT tumor cells.

Target cell proliferation was quantified over time using (A) an impedance-based assay (7316-3045) and (B) live cell imaging (SY5Y) after co-culture of target cells with CLDN6-CAR transfected T cells. Negative controls: CD19-CAR transfected T cells, untransduced T cells (UTD), tumor cells only; positive control: triton X-100 treated target cells. T cells were added at approximately 24 hours (vertical dotted line) at E:T ratios indicated in the legend. Lines depict the means of triplicate measurements.
